# Improving tuberculosis control: assessing the value of medical masks and case detection—a multi-country study with cost-effectiveness analysis

**DOI:** 10.1098/rsos.231715

**Published:** 2024-06-19

**Authors:** Dipo Aldila, Basyar Lauzha Fardian, Chidozie Williams Chukwu, Muhamad Hifzhudin Noor Aziz, Putri Zahra Kamalia

**Affiliations:** ^1^ Department of Mathematics, Faculty of Mathematics and Natural Sciences, Universitas Indonesia, Depok 16424, Indonesia; ^2^ Department of Mathematics, Wake Forest University, Winston-Salem, NC 27109, USA; ^3^ Faculty of Science, Institute of Mathematical Sciences, Universiti Malaya, Kuala Lumpur 50603, Malaysia

**Keywords:** tuberculosis, medical mask, case detection, reproduction number, transcritical bifurcation, sensitivity analysis

## Abstract

Tuberculosis (TB) remains a significant global health concern, necessitating effective control strategies. This article presents a mathematical model to evaluate the comparative effectiveness of medical mask usage and case detection in TB control. The model is constructed as a system of ordinary differential equations and incorporates crucial aspects of TB dynamics, including slow–fast progression, medical mask use, case detection, treatment interventions and differentiation between symptomatic and asymptomatic cases. A key objective of TB control is to ensure that the reproduction number, $R_{c}$ , remains below unity to achieve TB elimination or persistence if $R_{c}$ exceeds 1. Our mathematical analysis reveals the presence of a transcritical bifurcation when the $R_{c}=1$ signifies a critical juncture in TB control strategies. These results confirm that the effectiveness of case detection in diminishing the endemic population of symptomatic individuals within a TB-endemic equilibrium depends on exceeding a critical threshold value. Furthermore, our model is calibrated using TB yearly case incidence data per 100 000 population from Indonesia, India, Lesotho and Angola. We employed the bootstrap resampling residual approach to assess the uncertainty inherent in our parameter estimates which provides a comprehensive distribution of the parameter values. Despite a declining trend in new incidence, these four countries exhibit a reproduction number greater than 1, indicating persistent TB cases in the presence of ongoing TB control programmes. We employ the partial rank correlation coefficient in conjunction with the Latin hypercube sampling method to conduct a global sensitivity analysis of the $R_{c}$ parameter for each fitted parameter in every country. We find that the medical mask use is more sensitive to reduce $R_{c}$ compared with the case detection implementation. To further gain insight into the necessary control strategy, we formulated an optimal control and studied the cost-effectiveness analysis of our model to investigate the impact of case detection and medical mask use as control measures in TB spread. Cost-effectiveness analysis demonstrates that combining these interventions emerges as the most cost-effective strategy for TB control. Our findings highlight the critical importance of medical masks and their efficacy coupled with case detection in shaping TB control dynamics, elucidating the primary parameter of concern for managing the control reproduction number. We envisage our findings to have implications and be vital for TB control if implemented by policymakers and healthcare practitioners involved in TB control efforts.

## 1. Tuberculosis recent facts

Tuberculosis (TB) is an infectious disease caused by the bacterium *Mycobacterium tuberculosis* [1]. It primarily affects the lungs but can also target other parts of the body, such as the kidneys, spine and brain. TB is spread through the air when an infected person coughs, sneezes or talks, releasing tiny droplets containing the bacteria [2]. The infection is usually presen in two forms: latent TB infection (LTBI) and active TB disease [3]. In LTBI, the bacteria remain dormant within the body, and the infected individual does not experience symptoms or feel sick. However, they are at risk of developing active TB if their immune system weakens. Active TB, on the other hand, leads to noticeable symptoms like persistent cough, chest pain, fatigue, fever, night sweats and weight loss. It is essential to diagnose and treat active TB promptly, as it can be life-threatening if left untreated.

Preventive measures, such as vaccination (with the Bacillus Calmette–Guérin vaccine) for children and early identification and treatment of infected individuals, are crucial in controlling the spread of TB and reducing its impact on public health [4]. Effective treatment of TB involves a combination of antibiotics taken over a specific period, usually 6–9 months, to ensure complete eradication of the bacteria and reduce the risk of drug resistance [5]. In some cases of drug-resistant TB, treatment may require a more extended and challenging regimen. Medical masks, such as surgical masks or N95 respirators, can provide some level of protection against the transmission of TB, but they are not specifically designed as a primary preventive measure for TB [6]. While medical masks can help reduce the risk of inhaling large respiratory droplets that contain the TB bacteria, they are not entirely effective in preventing transmission.

The use of mathematical models by scientists in understanding the mechanisms of disease spread on a population scale has a long history. Many of these models have been inspired by the famous epidemic model developed by Kermack & McKendrick [7]. Subsequently, numerous mathematical models have been introduced to enhance our understanding of the spread of various well-known diseases, such as dengue [8,9], malaria [10,11], human immunodeficiency virus/acquired immune deficiency syndrome [12], pneumonia [13], coronavirus disease 2019 (COVID-19); [14,15]), TB [16,17] and many more. Recently, several mathematical models have been developed to gain a more specific understanding of the mechanisms underlying TB transmission on a population scale. Bhadauria *et al.* [18] studied the impact of isolation for TB cases in India using their Susceptible-Infected-Quarantine-Recovered (SIQR) model, in which they predicted that isolating half of the multidrug-resistant TB (MDR-TB) cases could lead to a substantial reduction in TB incidence by 2025, with a concurrent decline in estimated MDR-TB incidence. A mathematical model considering treatment was introduced by Ullah *et al.* [19]. Their parameter values were estimated using incidence data from Pakistan. They emphasized the significance of reducing the basic reproduction number to less than 1 as a pivotal strategy for eradicating TB epidemics and underscored the need to decrease treatment failure cases to reduce TB infectivity. The study also highlighted the effectiveness of isolating infective individuals through the reduction of the transmission coefficient. Okuonghae [20] performed a thorough mathematical analysis on a simplified stochastic TB disease model with case detection. The author found that the only disease persistence depends on the case detection parameter. Disease eradication showed an independent relationship with the case detection parameter. With the existence of pharmaceutical intervention in controlling the spread of TB, Liu *et al*. [21] proposed a stochastic TB model by incorporating the effect of antibiotic resistance. They found sufficient conditions (dependency between parameters on the reproduction number) for the extinction of TB from the population. An age-structured model for TB dynamics was constructed by Das & Kar [22]. It was found that the detection of LTBI could increase or decrease the reproduction number depending on the model parameter condition.

There are many options to prevent TB infection, with the most popular method being the use of vaccines. Vaccination aspects were incorporated into their delay-differential equation model by Zhang *et al.* [23]. The authors discovered that there is a minimum number of vaccinations such that vaccine intervention could effectively suppress the spread of TB. A different approach, as demonstrated by Yusuf & Abidemi [24], involves the use of an optimal control approach to model the impact of vaccines and treatments on TB dynamics. Besides vaccines, the intervention of treatment is also important for TB control programmes. Okuonghae [25] considered the impact of treatment with three different latently infected classes in their model. Furthermore, an innovative fractional-order stochastic differential equation model was introduced by Chukwu *et al.* [26] to analyse the impact of treatment on TB.

Despite these efforts, it is worth noting that not many mathematical models have considered the use of medical masks and case detection, similar to the strategies used during the COVID-19 pandemic, as simple and easy-to-implement prevention strategies for TB. Therefore, our proposed model in this article will incorporate two different interventions for preventing and controlling the spread of TB, namely the use of medical masks and case detection. The model was developed using a five-dimensional system of ordinary differential equations (ODE). Mathematical analysis and numerical experiments were conducted to demonstrate the long-term behaviour of the proposed model.

The layout of our article is organized into several key sections, each addressing distinct aspects of our research: §1 provides an overview of recent facts about TB and review previous mathematical models relevant to our study; §2 outlines our model assumptions, construction methodology and details the parameter estimation process; §3 delves into the dynamical analysis of our model, focussing on equilibrium points, the reproduction number and bifurcation analysis to understand the underlying TB dynamics of our model. Moving forward, §4 is dedicated to global sensitivity analysis (GSA), where we explore the impact of medical masks and case detection on controlling the reproduction number of TB; §5 presents the results of our optimal control simulations and conducts a cost-effectiveness analysis to evaluate the efficacy and efficiency of various TB control strategies; and §6 encapsulates our conclusions, summarizing key findings, implications and avenues for future research. Additionally, we provide appendices containing proofs and visualizations of theorems presented throughout the manuscript, offering supplementary information to enhance the understanding and rigour of our study.

## 2. Mathematical model construction and parameter estimation

### 2.1. Model construction

Let the human population be divided into five compartments, namely susceptible ( S ), exposed/latent ( E ), infected asymptomatic, undetected and untreated ( $I_{1}$ ), infected symptomatic, detected and treated ( $I_{2}$ ) and recovered ( R ). Therefore, the total human population is given by:


$$N=S+E+I_{1}+I_{2}+R.$$

To construct the mathematical model for TB transmission in this research, several important assumptions need to be declared first:

— *case detection*: case detection is a crucial component of TB control efforts aimed at identifying and diagnosing individuals with active TB disease. Effective case detection is essential for initiating prompt treatment, preventing further spread of the disease and ultimately reducing the burden of TB [27]. Based on this importance, we include the case detection effort $\left(u_{1}\right)$ in our model to find symptomatic undetected individuals in the field. Hence, $u_{1}I_{1}$ represents the newly detected symptomatic TB-infected individuals;
— *effect of medical mask use*: with the escalation of respiratory diseases, including TB, the use of medical masks emerges as one of the most prevalent and pragmatic non-pharmaceutical interventions aimed at diminishing the risk of infection and disease transmission. While the effectiveness of wearing medical masks in combating diseases remains a topic of debate [28–30], numerous pre-COVID-19 pandemic studies highlight the potential efficacy of medical mask usage among TB-active patients in curtailing the spread of TB. In three human studies conducted in healthcare settings, a reduction in TB cases was observed among the participants who used the medical masks [31–33]. The findings were also consistent with an animal study by Dharmadhikari *et al.* [34]. The study reported that 56% decreased risk of TB transmission in a group of guinea pigs when exposed to air from active TB patients who wore masks. To model the impact of medical mask use, let us denote β and $u_{2}$ as the TB successful infection rate and rate of medical mask use, respectively. Furthermore, it is assumed that individuals in compartment $I_{2}$ cannot transmit TB to others because they are presumed to adhere to recommendations to reduce close contact with others, either by maintaining distance, isolation or quarantine. Hence, we have $u_{2}I_{1}$ represent the proportion of infected individuals who use medical masks, while $(1-u_{2})I_{1}$ are those who do not. Hence, the total new infection caused by infected individuals who do not use medical masks is given by $\beta (1-u_{2})I_{1}S$ . Furthermore, we assume that the use of a medical mask may reduce the successful infection rate with an efficacy of ξ . The more effective the medical mask, the larger the value of ξ . Hence, the total number of new infections caused by infected individuals who use medical masks is given by $(1-\xi )\beta u_{2}I_{1}S$ . Therefore, the total number of new infections, $\Lambda (S,I_{1})$ caused by $I_{1}$ is given by:

$$\Lambda (S,I_{1})=\beta (1-u_{2})I_{1}S+(1-\xi )\beta u_{2}I_{1}S=(1-\xi u_{2})\beta SI_{1};$$

— *slow–fast progression*: TB infection can exhibit different progression patterns, including slow and fast progression [35]. In slow progression, the infection advances gradually over an extended period. Individuals with slow-progressing TB may not show noticeable symptoms for a significant period after being exposed to the bacteria. Latent TB may later progress to active TB disease under certain conditions, such as a weakened immune system. On the other hand, fast progression refers to a more rapid development of active TB disease after exposure to the bacteria. Individuals with fast-progressing TB may experience symptoms relatively soon after being infected. Based on this, we assume that the total number of new infections given by $\Lambda (S,I_{1})$ may experience slow progression with a probability of p or fast progression with a probability of q . Note that p+q=1 . Hence, the proportion of newly infected individuals who experience slow progression is given by:

$$p\,\Lambda \,\left(S,I_{1}\right)=p\,\left(1-\xi \,u_{2}\right)\,\beta \,S\,I_{1},$$

while for fast progression is given by


$$q\,\Lambda \,\left(S,I_{1}\right)=q\,\left(1-\xi \,u_{2}\right)\,\beta \,S\,I_{1}.$$

Using the above assumptions, the model construction is given as follows: we assume that the recruitment rate is always constant with a value of δ . Under the impact of medical mask use, the number of susceptible individuals may decrease owing to a new infection by $I_{1}$ individual, given by $(1-\xi u_{2})\beta SI_{1}$ . Owing to slow–fast progression, a proportion of newly infected individuals will experience slow progression $\left(p(1-\xi u_{2})\beta SI_{1}\right)$ and enter the E compartment, while the rest will experience fast progression $\left(q(1-\xi u_{2})\beta SI_{1}\right)$ and move to the $I_{1}$ compartment. Furthermore, it is assumed that latent individuals can undergo an increase in infection status, becoming infectious and exhibiting symptoms, thus requiring treatment. We use ϵ to represent this phenomenon, which allows the transition from E to $I_{2}$ . Without any early case detection, latent individuals will experience TB progression and become infected. Hence, there is a transition rate from E to $I_{1}$ owing to infection progression, denoted by θ .

Owing to case detection for the symptomatic individual $I_{1}$ , we have a transition from $I_{1}$ to $I_{2}$ with a rate of $u_{1}$ . Both $I_{1}$ and $I_{2}$ may recover from TB with a rate of recovery given by $k_{1}$ and $k_{2}$ , respectively. Since $I_{2}$ gets an intensive treatment, we have $k_{2}>k_{1}$ . In addition to natural death occurring at a rate μ in each compartment, there is a death rate specifically due to TB for compartments $I_{1}$ and $I_{2}$ with rates $d_{1}$ and $d_{2}$ , respectively.

Hence, the TB model with interventions such as case detection and medical masks is given by:


(2.1) $$\frac{dS}{dt}=\delta -(1-u_{2}\xi )\beta SI_{1}-(\mu )S,$$

(2.2) $$\begin{array}{rl} \frac{dE}{dt} & =p(1-u_{2}\xi )\beta SI_{1}-(\theta +\epsilon +\mu )E, \end{array}$$

(2.3) $$\begin{array}{rl} \frac{dI_{1}}{dt} & =q(1-u_{2}\xi )\beta SI_{1}+\theta E-(u_{1}+k_{1}+d_{1}+\mu )I_{1}, \end{array}$$

(2.4) $$\begin{array}{rl} \frac{dI_{2}}{dt} & =\epsilon E+u_{1}I_{1}-(\mu +d_{2}+k_{2})I_{2}, \end{array}$$

(2.5) $$\begin{array}{rl} \frac{dR}{dt} & =k_{1}I_{1}+k_{2}I_{2}-\mu R, \end{array}$$

with non-negative initial conditions. Let


$$\Omega =\left\{(S,E,I_{1},I_{2},R)\in R_{+}^{5}|0\leq N\leq \max\left\{N(0),\frac{\delta }{\mu }\right\}\right\},$$

defined as the invariant region for equations (2.1)–(2.5). As long as the initial conditions are in Ω , the solution of the above system will always remain in Ω . Interested readers can see appendix A for the proof.

### 2.2. Estimating model parameters using data fitting

Before conducting simulations for the optimal control problem in §6, we performed parameter estimation for our model using yearly new incidence data per 100 000 individuals from four different countries: Indonesia, India, Angola and Lesotho. The data, starting from 2000 to 2020, were obtained from the World Bank [36].

We aimed to find the best-fit parameter and best-fit initial conditions of our model such that the Euclidean distance between the incidence data and model output simulation was minimized. Since the data is the yearly new incidence data, we fitted the data with the newly detected incidence of TB both from E and $I_{1}$ compartments, i.e. ϵE and $u_{1}I_{1}$ . Particularly, the following cost function was minimized:


(2.6) $$C=\sum_{i=0}^{21}{\left((\epsilon E_{i}+u_{1}{I_{1}}_{i})-{\text{data}}_{i}\right)}^{2},$$

where 21 is the number of data points collected from the years 2000 to 2020 for each country. Other parameters were held constant as follows:

– *the recruitment rate (* δ *) and the natural death rate (* μ *)*: from equations (2.1)–(2.5), the dynamic of the total human population is given by:

$$\frac{dN}{dt}=\delta -\mu (S+E+I_{1}+I_{2}+R)-d_{1}I_{1}-d_{2}I_{2}\leq \delta -\mu N.$$

Hence, if we assume that $d_{1}$ and $d_{2}$ are relatively small, then the total population can be assumed to be constant. Hence, we have δ=μN . Given that the incidence data used for parameter estimation is presented as the incidence rate per 100 000 people [36], it follows that our population size, denoted as N , is equivalent to 100 000. Furthermore, since the average human life expectation is between 66.8 years in 2000 to 73.4 in 2019 [37], then we assume that $\mu =\frac{1}{72}$ ;

— *medical mask efficacy (* ξ *)*: the use of surgical face masks on patients with MDR-TB has demonstrated a significant reduction in transmission, providing an additional measure to mitigate the spread of TB from infectious individuals [34]. Hence, from the same reference, we choose ξ=56% ;
— *the TB latent progression to active TB (* θ *)*: reactivation is the transition of a subclinical latent infection into active TB disease. Consequently, individuals with LTBI serve as a significant reservoir for the emergence of new active TB cases. According to [38], it takes approximately 2 years for an individual to progress from latent TB to active TB. Hence, we assume $\theta =\frac{1}{2}$ ;
— *the recovery rate (* $k_{1}$ *and* $k_{2}$ *)*: most individuals with TB disease typically require a minimum of 6–12 months of TB treatment for a complete cure [39]. Hence, we assume $k_{2}=1$ . Since $k_{1}<k_{2}$ , then we assume $k_{1}=0.5$ ; and
— *death rate owing to TB* $(d_{1}$ *and* $d_{2}$ *)*: it is assumed that the death rate due to TB for $I_{1}$ and $I_{2}$ is 0.01 .

**Figure 1.**
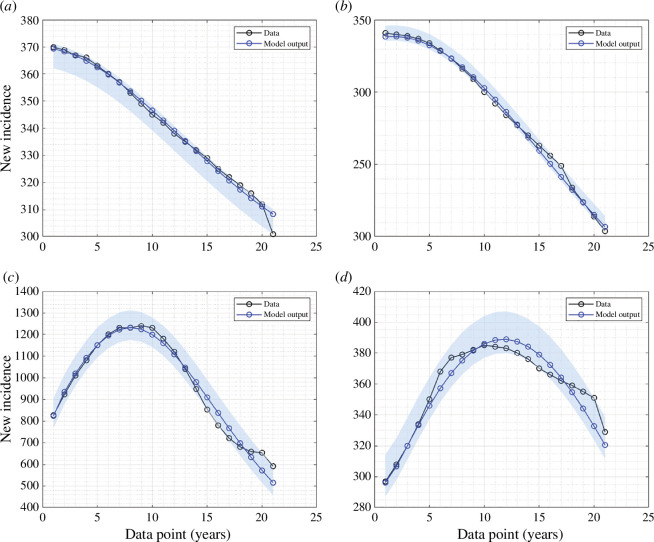
Fitted data of new incidence cases for (*a*) Indonesia, (*b*) India, (*c*) Lesotho and (*d*) Angola. The solid black and blue lines represent the real data and simulated data, respectively.

The other parameters, namely, the infection rate β , the proportion of slow–fast progression p and q , case detection rate $u_{1}$ , the proportion of medical mask use $u_{2}$ , and the transition to symptomatic from latent individual ϵ , were estimated together with the initial conditions. Mathematically, it can be written as


$$C(\beta^{*},p^{*},q^{*},\epsilon^{*},u_{1}^{*},u_{2}^{*},X^{*}(0))=\min_{\Delta }C(\beta ,p,q,\epsilon ,u_{1},u_{2},X(0)),$$

where X(0) is the set of initial condition of equations (2.1)–(2.5), and Δ is the set of admissible range of parameter values. In this study, we employ the fmincon toolbox to estimate the parameters of our model, as described byequations (2.1)–(2.5). fmincon is a powerful optimization tool in MATLAB, typically used for solving constrained nonlinear optimization problems. In our adaptation of fmincon for ODEs, we formulate the parameter estimation task as an optimization problem where the objective function represents the discrepancy between model predictions and observed data, subject to any pertinent constraints. We iteratively refine the model parameters by optimizing this objective function until a satisfactory fit to the data is achieved. Additionally, to assess the uncertainty inherent in our parameter estimates and provide a comprehensive distribution of parameter values, we employ the bootstrap resampling residual approach [40,41] across all estimation results for four distinct countries. This approach allows us to generate multiple parameter sets by resampling residuals, providing insight into the variability and robustness of our model across different datasets and scenarios. The fitting results for the incidence data of four different countries are given in figure 1, and the best-fit parameter values are listed in table 1.

**Table 1.** The best-fit parameters and best-fit initial conditions for the fitted curves in figure 1.

country	$\beta (10^{-5})$	p	q	ϵ	$u_{1}$	$u_{2}$	S(0)	E(0)	$I_{1}(0)$	$I_{2}(0)$	R(0)	$R_{0}$
Indonesia	3.356	0.885	0.115	0.121	0.151	0.5	34 152	1546	1208	2179	57	2.907
India	1.752	0.899	0.101	0.087	0.102	0.5	58 975	2075	1549	5543	523	1.711
Lesotho	2.061	0.883	0.117	0.155	0.198	0.5	80 871	1586	2921	3012	475	1.967
Angola	1.251	0.898	0.102	0.045	0.13	0.5	93 995	1821	1649	3027	621	1.247

In the next section, we provide a complete mathematical analysis of the model, including the existence and local stability of the equilibrium points of equation (2.1) as well as the control reproduction number $(R_{c}).$

## 3. Dynamical analysis

### 3.1. Disease-free equilibrium point $\left(E_{1}\right)$

The disease-free equilibrium (DFE) of equations (2.1)–(2.5) was obtained by letting the right-hand side of equations (2.1)–(2.5) to zero and setting $I_{1}=I_{2}=0$ . Hence, we have:


(3.1) $$E_{1}=(S,E,I_{1},I_{2},R)=\left(\frac{\delta }{\mu },0,0,0,0\right).$$

### 3.2. Control reproduction number $\left(R_{c}\right)$

**Theorem 1.** *The control reproduction number of* equations (2.1)–(2.5) *, denoted by* $R_{c}$ *is given by*:


(3.2) $$R_{c}=\frac{\delta \beta \left(1-u_{2}\xi \right)(\theta +q(\epsilon +\mu ))}{\mu (\theta +\epsilon +\mu )(u_{1}+k_{1}+d_{1}+\mu )}.$$

Refer to appendix B for the derivation of the control reproduction number.

It is common in many epidemiological models [42–44] for the reproduction number to determine whether the disease may die out or persist in the population. In many cases, authors have found that the disease can go extinct if the reproduction number is less than 1, and it always has a chance to persist if the basic reproduction number is larger than 1. In the next theorem, we use the results by van den Driessche & Watmough [45] to show the local stability criteria of the equilibrium $E_{1}$ .

**Theorem 2.** *The disease-free equilibrium* $E_{1}$ *of* equations (2.1)–(2.5) *of equations (2.1)-(2.5) is locally asymptotically stable if* $R_{c}<1$ *, and unstable if* $R_{c}>1$ .

See appendix C for the complete proof of Theorem 2.

### 3.3. Global stability of the disease-free equilibrium

Using the approach in [46], we prove the existence of global asymptotic stability (GAS) for the DFE of our TB model. First, we rewrite equations (2.1)–(2.5) as follows:


(3.3) $$\left\{ \begin{array}{lll} \frac{dX}{dt} & = & F(X,I),\\ \frac{dI}{dt} & = & G(X,I),\;\;G(X,0)=0, \end{array}\right.$$

in which $X=(S,R)\in R^{2}$ and $I=(E,I_{1},I_{2})\in R^{3}.$ Note that the variables X and I represent un-infectious and infectious TB individuals, respectively. For the model to be GAS at $E_{1},$ it needs to satisfy the following conditions as stated in [46], that is:

– local stability is guaranteed at $E^{0}$ whenever $R_{0}<1;$

– at $\frac{dX}{dt}=F(X_{0},0)$ , the DFE is globally asymptotically stable; and
–
$G(X,I)=AI-\hat{G}(X,I),\hat{G}(X,I)\geq 0$ for (X,I)∈Ω, where $X_{0}=E_{1},\;A=D_{I}G(E_{0})$ is a Metzler matrix, and Ω is our TB-model biologically feasible region.

**Theorem 3.** *Let* t>0, *then the disease-free equilibrium* $E_{1}$ *is GAS stable if* $R_{0}$ .

See appendix D for the complete proof of Theorem 3.

### 3.4. Endemic equilibrium point

Taking the right-hand side of equations (2.1)–(2.5) equal to 0 and solving it with respect to each variable, then we have the endemic equilibrium point of equations (2.1)–(2.5) given by:


(3.4) $$E_{2}=(S^{†},E^{†},I_{1}^{†},I_{2}^{†},R^{†}),$$

where


$$\begin{array}{l} S^{†}=\frac{\delta }{\mu }\frac{1}{R_{c}},\\ E^{†}=\frac{p\delta }{\theta +\epsilon +\mu }\left(1-\frac{1}{R_{c}}\right),\\ I_{1}^{†}=\frac{\delta (\theta +q(\epsilon +\mu )}{\left(\theta +\epsilon +\mu )(u_{1}+k_{1}+d_{1}+\mu \right)}\left(1-\frac{1}{R_{c}}\right),\\ I_{2}^{†}=\frac{\left(\left(u_{1}+p\left(k_{1}+d_{1}+\mu \right)\right)\epsilon +u_{1}\left(q\mu +\theta \right)\right)\delta }{\left(u_{1}+k_{1}+d_{1}+\mu \right)\left(\theta +\epsilon +\mu \right)\left(\mu +d_{2}+k_{2}\right)}\left(1-\frac{1}{R_{c}}\right),\\ R^{†}=\frac{k_{1}I_{1}^{†}+k_{2}I_{2}^{†}}{\mu }. \end{array}$$

Based on the expression of $E_{2}$ above, we have the following theorem.

**Theorem 4.** *There always exists a unique endemic-equilibrium point* $E_{2}$ *of system (1) if* $R_{c}>1$ *.*

*Proof*. The proof of this theorem can be directly seen from the expressions of $S^{†},E^{†},I_{1}^{†},I_{2}^{†},R^{†}$ . Each of these expressions should be positive. For any positive parameters, we will always have $S^{†}>0.$ On the other hand, $E^{†},I_{1}^{†},I_{2}^{†}$ will be positive only if $R_{c}>1$ . Finally, $R^{†}$ is always positive since the total population is less than $\frac{\delta }{\mu }$ . Hence, the proof is completed.

### 3.5. Non-existence of backward bifurcation

**Theorem 5.** *System equations (2.1)–(2.5) always exhibits a transcritical bifurcation at* $R_{c}=1$ *.*

We use Castillo–Song bifurcation theorem [47] to proof Theorem 5. See appendix E for the complete proof of Theorem 5.

Based on Theorem 5, we can observe that the backward bifurcation phenomenon never occurs in our proposed TB model as described in equations (2.1)–(2.5). On the other hand, as per Theorem 3, we know that the disease-free equilibrium is globally asymptotically stable when $R_{c}<1.$ Therefore, it is reasonable to hypothesize that the endemic equilibrium point is globally asymptotically stable when $R_{c}>1$ . We leave the proof of this statement as an open problem for readers who may be interested.

### 3.6. Impact of medical mask and case detection on the endemic of tuberculosis

#### 3.6.1. Comparison of the control and the basic reproduction number

In a simple case of no control intervention $u_{1}=0,u_{2}=0$ , then we can reduce the control reproduction $R_{c}$ in the following basic reproduction number:


(3.5) $$R_{0}=\frac{\delta \beta (\theta +q(\epsilon +\mu ))}{\mu (\theta +\epsilon +\mu )(k_{1}+d_{1}+\mu )}.$$

Since for all positive parameters, we have


$$R_{0}-R_{c}=\frac{\beta \left(\left(\epsilon +\mu \right)q+\theta \right)\delta }{\mu \left(\theta +\epsilon +\mu \right)\left(k_{1}+d_{1}+\mu \right)}>0,$$

we have the following remarks.

*Remark 1*. Any positive intervention of medical mask use or case detection will always be successful in reducing the basic reproduction number $R_{0}$ .

#### 3.6.2. Impact of medical mask and case detection to $R_{c}$

The expression of $R_{c}$ can be expressed as a function of $R_{0}$ as follows:


(3.6) $$R_{c}=R_{0}\times F,$$

where $F=\frac{k_{1}+d_{1}+\mu }{u_{1}+k_{1}+d_{1}+\mu }\times (1-u_{2}\xi )<1$ is the reduction factor of $R_{0}$ owing to case detection $u_{1}$ and medical mask use $u_{2}$ . Based on this, we have the following remark.

*Remark 2*. The following remark is the direct interpretation of expression in equation (3.6).

– For a special case where the case detection rate tends to ∞ , then we have:

$$\lim_{u_{1}\to \infty }R_{c}=0,$$

which implies a massive intervention in case detection can reduce the reproduction number significantly.

– For a special case when all individuals use a medical mask $\left(u_{2}=1\right)$ , then we have:

$$\lim_{u_{2}\to 1}R_{c}=R_{0}\times \frac{k_{1}+d_{1}+\mu }{u_{1}+k_{1}+d_{1}+\mu }\times (1-\xi ),$$

which implies that using higher quality medical masks (ξ→1) can lead to a more efficient reduction of the control reproduction number.

#### 3.6.3. Impact of case detection to $I_{1}^{†}$ and $I_{2}^{†}$

To analyse the impact of case detection and medical mask use on the size of $I_{1}$ and $I_{2}$ at the endemic equilibrium, we differentiate $I_{1}^{†}$ and $I_{2}^{†}$ with respect to $u_{1}$ and $u_{2}$ . Derivation of $I_{1}^{†}$ respect to $u_{1}$ and $u_{2}$ gives:


$$\begin{array}{l} \frac{\partial I_{1}^{†}}{\partial u_{1}}=-\frac{\delta (\theta +q(\mu +\epsilon ))}{(\theta +\mu +\epsilon )(k_{1}+d_{1}+u_{1}+\mu {)}^{2}}<0,\\ \frac{\partial I_{1}^{†}}{\partial u_{2}}=-\frac{\mu (1-\xi )}{(1-\xi u_{2}{)}^{2}\beta }<0. \end{array}$$

For any value of $u_{1}>0$ and $u_{2}\in [0,1]$ , the signs of $\frac{\partial I_{1}^{†}}{\partial u_{1}}$ and $\frac{\partial I_{1}^{†}}{\partial u_{2}}$ are always negative. This indicates that the size of $I_{1}^{†}$ will always reduce whenever case detection or medical mask use is implemented for TB control. Furthermore, since $u_{1}$ only appears in the denominator, both $u_{1}$ and $u_{2}$ have a significant impact on the change in the size of $I_{1}^{†}$ for an early implementation, as $\lim_{u_{1}\to \infty }\frac{\partial I_{1}^{†}}{\partial u_{1}}=0$ and $\lim_{u_{2}\to 1}\frac{\partial I_{1}^{†}}{\partial u_{2}}=0$ . With these results, we have the following remark.

*Remark 3*. Implementation of case detection is always successful in reducing the number of asymptomatic infected individuals at the TB-endemic equilibrium.

On the other hand, we have


$$\begin{array}{lll} \frac{\partial I_{2}^{†}}{\partial u_{1}} & = & \frac{\mu \delta {\left(q\left(\epsilon +\mu \right)+\theta \right)}^{2}\left(1-\xi u_{2}\right)\left(k_{1}+d_{1}+\mu \right)\beta -\mu^{2}{\left(u_{1}+k_{1}+d_{1}+\mu \right)}^{2}\left(\theta +\epsilon +\mu \right)\left(q\mu +\epsilon +\theta \right)}{\mu \left(\theta +\epsilon +\mu \right){\left(u_{1}+k_{1}+d_{1}+\mu \right)}^{2}\beta \left(1-\xi u_{2}\right)\left(q\left(\epsilon +\mu \right)+p\theta \right)\left(\mu +d_{2}+k_{2}\right)},\\ \frac{\partial I_{2}^{†}}{\partial u_{2}} & = & -\frac{\left(\left(\left(k_{1}+d_{1}+\mu \right)p+u_{1}\right)\epsilon +\left(\theta +\mu \right)u_{1}\right)\left(1-\xi \right)\mu }{{\left(1-\xi u_{2}\right)}^{2}\beta \left(\epsilon q+\mu +\theta \right)\left(\mu +d_{2}+k_{2}\right)}<0. \end{array}$$

We can see that $\frac{\partial I_{2}^{†}}{\partial u_{2}}$ is always negative. Therefore, increasing medical mask use in the population will reduce the size of $I_{2}^{†}$ . However, $\frac{\partial I_{2}^{†}}{\partial u_{1}}$ is not always negative. Solving $\frac{\partial I_{2}^{†}}{\partial u_{1}}=0$ with respect to $u_{1}$ gives us $u_{1}^{*}$ as a critical value at which the sign of $\frac{\partial I_{2}^{†}}{\partial u_{1}}$ changes from positive to negative. With these results, we have the following remark.

*Remark 4*. The implementation of medical masks consistently reduces the number of symptomatic infected individuals. Additionally, there exists a critical value for case detection, beyond which the implementation of case detection successfully reduces the number of symptomatic infected individuals in the context of TB-endemic equilibrium.

**Figure 2.**
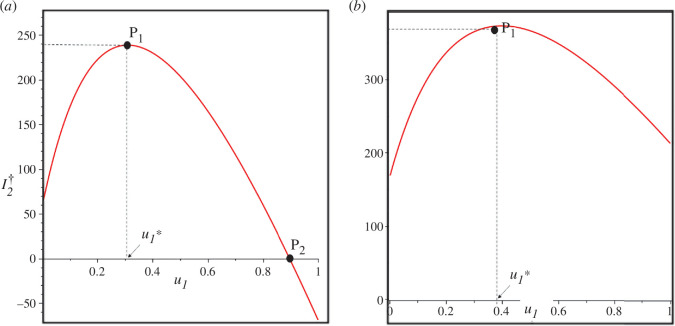
Curve of $I_{1}^{†}$ as a function of $u_{1}$ using Lesotho and Indonesia data in panels (*a*) and (*b*), respectively. $P_{1}$ is the turning point of $I_{2}^{†}$ , while $P_{2}$ is $u_{2}$ when $R_{c}=1$ . When $u_{1}<u_{1}^{*}$ , then $I_{1}^{†}$ increases as $u_{1}$ increases. The critical value $u_{1}^{*}$ for Lesotho and Indonesia is 0.308 and 0.398, respectively. TB-endemic equilibrium only exists when $u_{1}<P_{2}$ .

The illustration of the aforementioned remarks can be seen in figure 2 using estimation results for Lesotho (panel *a*) and for Indonesia’s data (panel *b*). In both panels, it is observed that there exists a minimum value $u_{1}=u_{1}^{*}$ such that only when $u_{1}>u_{1}^{*}$ an increase in the case detection rate can reduce the number of individuals symptomatic with TB ( $I_{2}$ ) at the endemic equilibrium. Conversely, if $u_{1}<u_{1}^{*}$ , an increase in the case detection rate raises the value of $I_{2}$ at the endemic equilibrium. Furthermore, $P_{2}$ represents the value of $u_{1}$ such that $R_{0}=1$ . Therefore, for the case using Lesotho’s data, it is evident that the implementation of case detection can be relied upon to eliminate TB cases in Lesotho, specifically when $u_{1}>0.896$ . On the other hand, for the data from Indonesia, it is observed that there is no point $P_{2}$ within the range of $u_{2}\in [0,1]$ . This implies that the case detection intervention cannot eliminate TB in Indonesia.

In this section, we have discussed the dynamic properties of the model presented in equations (2.1)–(2.5). These dynamic properties include that the model in equations (2.1)–(2.5) always has a stable TB-free equilibrium point for $R_{c}<1$ , and it becomes unstable when $R_{c}>1$ . The point $R_{c}=1$ serves as a bifurcation point where the stability of the TB-free equilibrium point changes, marking the emergence of the endemic equilibrium point. A new TB-endemic equilibrium point emerges and is always stable under conditions where $R_{c}>1$ . For further visualization of these results, such as bifurcation diagrams and one-parameter sensitivity analysis, readers can refer to appendix F.

In the following section, we will provide a study on parameter sensitivity, including GSA using the partial rank correlation coefficient (PRCC) and Latin hypercube sampling (LHS), as well as two-parameter sensitivity analysis to examine the influence of vaccine efficacy (ξ) and the tendency for fast infection progression (q) on the intensity of medical mask distribution and case detection in TB control.

## 4. Sensitivity analysis

### 4.1. Global sensitivity analysis

**Figure 3.**
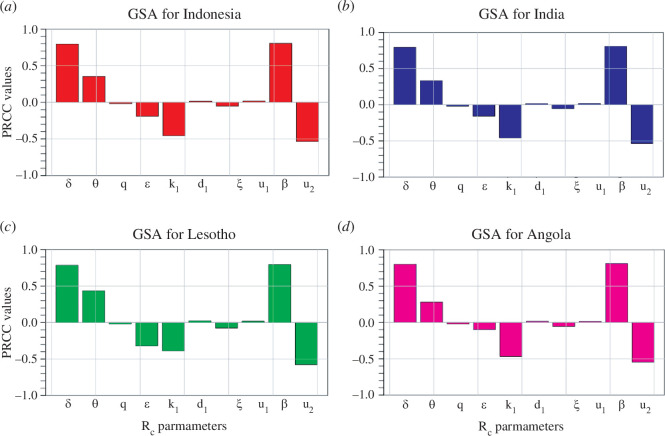
Plots showing the GSA on *$R_{c}$* parameters excluding *μ* for (*a*) Indonesia (*b*) India, (*c*) Lesotho and (*d*) Angola.

This subsection is devoted to carrying out the GSA of our TB model. GSA is a process of investigating uncertainty analysis in a model output parameter given a model input factor over an entire range of interest. In addition, some advantages of using GSAs are: (i) it considers all the input factors/parameters which are varied simultaneously while evaluating parameter sensitivity over the entire range of each input factor/time frame under investigation; (ii) it helps to identify model parameters that are more sensitive to infection threshold, which may be the infectious disease classes or control reproduction number as in our TB model; and (iii) it also assesses the variability in model predictions, usually introduced by uncertainty in the parameter values. Knowing this information is relevant for policymaking in the management of the spread of both infectious and non-infectious diseases. To determine the variability in model parameters contained in control reproduction number, $R_{c}$ , we use a combination of LHS and the PRCC technique [48,49]. Parameters with PRCC values above 0.5 or below −0.5 are the most significant or have strong correlations, which could be positive or negative, respectively [48,49]. This method looks at the relationship between $R_{c}$ and all its parameters. In this analysis, we use R software with 1000 simulations per run, and the resulting PRCC values indicate the effect of the parameters on the control reproduction number generated. These numerical results showing the PRCC for each parameter are shown as a Tornado plot in figure 3 for Indonesia, India, Lesotho and Angola, respectively.

In figure 3*a*, which represents the GSA results for Indonesia, the parameters $\delta ,\beta ,u_{2}$ and θ are the most sensitive. The infection rate β is positively correlated to $R_{c}.$ This implies that it contributes to increasing the number of infectious individuals, which leads to more humans infected with TB in Indonesia. Similar results apply to δ and θ. By contrast, the control parameter $u_{2}$ has a negative correlation, which thus implies that effective mask usage reduces $R_{c}$ and, in turn, reduces the number of symptomatic infected TB individuals. A clear look at the results presented in figure 3*b*–*d* indicates that similar results are obtained for the TB transmission path in India, Lesotho and Angola, respectively. Many epidemic models in the literature have applied similar analyses to, for instance, malaria [50,51], pneumonia [52], COVID-19 [53,54], listeriosis [55] and many others.

### 4.2. Effect of medical mask efficacy and fast-progression on the intensity of medical mask and case detection: a two-parameter sensitivity analysis

**Figure 4.**
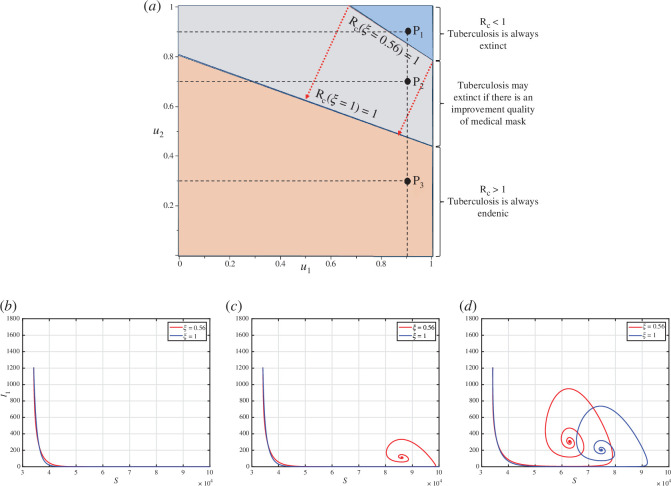
A two-parameter sensitivity analysis of $R_{c}$ respect to $u_{1}$ and $u_{2}$ under the impact of ξ given in panel (*a*). The orange region represents an $R_{c}$ domain that is always larger than 1, while blue is always smaller than 1, independent of the value of ξ . The grey region represents the area of $R_{c}$ that may change from greater than 1 to smaller than 1 as ξ increases. Panels (*b)* to *(d*), show the dynamics of S and $I_{1}$ for sample points $P_{1}$ , $P_{2}$ and $P_{3}$ , respectively.

In this subsection, we conducted a two-parameter sensitivity analysis on $R_{c}$ with respect to the control variables $u_{1}$ and $u_{2}$ , as well as the quality of medical mask parameter ξ . We considered two different values of ξ to represent the quality of a medical mask: ξ=0.56 and ξ=1 . The value of ξ=0.56 represents a condition that the medical mask offers 56% protection against the disease. On the other hand, if ξ=1 represents a perfect quality of medical mask. All other parameter values are based on the best-fit parameter for Indonesia, which is shown in table 1. The results are shown in figure 4. Based on figure 4*a*, an increase in the values of $u_{1}$ and $u_{2}$ increases the possibility of the value of $R_{c}$ becoming smaller than 1. This is in line with the analysis as depicted in appendix F.

As mentioned before, the larger the values of $u_{1}$ and $u_{2}$ , the greater the possibility that the value of $R_{c}$ becomes smaller than 1. The orange-coloured region represents the $R_{c}$ region, consistently exceeding 1, while the blue-coloured region signifies the $R_{c}$ region consistently remaining below 1, despite a medical mask efficacy of only 56%. The grey area represents the range where $R_{c}$ could change from being greater than 1 (when ξ=0.56 ) to being less than 1 (when ξ=1 ). In other words, efforts related to the case detection rate $\left(u_{1}\right)$ or the proportion of individuals using medical masks $\left(u_{2}\right)$ can be minimized if the quality of the medical mask is improved. These findings also indicate that enhancing medical mask quality can indirectly contribute to reducing the required case detection rate, thereby controlling TB in the field.

To illustrate the influence of changes in ξ on the effectiveness of $u_{1}$ and $u_{2}$ regarding the variation of $R_{c}$ values, we selected three sample points, namely $P_{1}$ , $P_{2}$ and $P_{3}$ , as shown in figure 4*b–d*. Point $P_{1}$ is located in the blue region, where TB can be eradicated from the population irrespective of the quality of medical masks, whether it is 56% or 100%. Figure 4*b* illustrates that the dynamic of solutions consistently converges towards the TB-free equilibrium. On the contrary, Point $P_{3}$ is situated in the orange area, where TB cannot be eliminated from the population regardless of the quality of the medical mask. Figure 4*d* demonstrates that the dynamic of S and $I_{1}$ consistently tends towards the TB-endemic equilibrium. However, it is evident that a higher quality of medical mask results in a smaller endemic size of $I_{1}$ . Point $P_{2}$ is located in the grey area, where TB elimination depends on the quality of the medical mask. If the medical mask efficacy is only 56%, then TB will persist in the population, as indicated by the red curve in figure 4*b* tending towards the TB-endemic equilibrium. Conversely, with a medical mask efficacy of 100%, TB can be eliminated from the population, as depicted by the blue curve in figure 4*b* converging towards the TB-free equilibrium.

**Figure 5.**
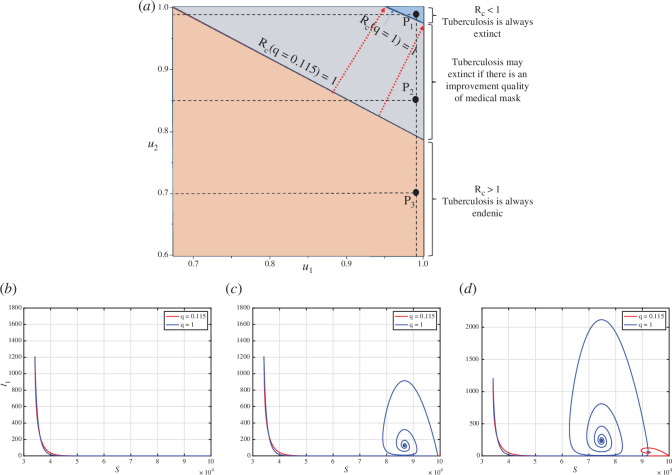
A two-parameter sensitivity analysis of $R_{c}$ with respect to $u_{1}$ and $u_{2}$ is presented under the influence of the parameter q (depicted in panel *a*). The orange region denotes the $R_{c}$ domain consistently exceeding 1, while the blue region consistently remains below 1, irrespective of the value of q . The grey region represents a zone where $R_{c}$ may either fall below or surpass 1, contingent upon the value of q . Panels (*b*) to (*d*) illustrate the dynamic behaviours of S and $I_{1}$ for the sample points $P_{1}$ , $P_{2}$ and $P_{3}$ , respectively.

Next, we analyse the sensitivity of $u_{1}$ and $u_{2}$ with respect to the value of $R_{c}$ , using different values of the proportion of fast progression ( q ). A larger value of q indicates that more people proceed directly to active TB after their initial infection. Therefore, it is evident from figure 5*a* that a higher value of q will require more intense implementation of case detection and medical mask use to eliminate TB from the population.

We present an illustration of the dynamics of S and $I_{1}$ based on various combinations of $u_{1}$ , $u_{2}$ and q . In the case of the combination in $P_{1}$ (as shown in figure 5*b*), both the dynamics of S and $I_{1}$ tend towards the TB-free equilibrium point. A smaller value of q accelerates the convergence of both S and $I_{1}$ towards the TB-free equilibrium point. Similarly, in figure 5*d*, both dynamics tend towards the TB-endemic equilibrium point, as $R_{c}>1$ at $P_{3}$ . However, it is important to note that $P_{2}$ (shown in figure 5*b*) does not always result in $R_{c}<1$ . For instance, when q=0.115 , the combination of $u_{1}$ and $u_{2}$ at $P_{2}$ yields $R_{c}<1$ , leading to dynamics that approach the TB-free equilibrium point (see the red curve). Conversely, when q=1 , the combination of $u_{1}$ and $u_{2}$ at $P_{2}$ results in $R_{c}>1$ , causing the dynamics of S and $I_{1}$ tend towards the TB-endemic equilibrium point (see the blue curve).

## 5. Optimal control model of case detection and medical mask

### 5.1. Optimal control characterization

As mentioned in the previous analysis, it is clear that a more substantial intervention in case detection and medical mask usage will significantly reduce both the size of the control reproduction number and the size of the infected compartment in the TB-endemic equilibrium. However, the more extensive the intervention, the higher the cost. Therefore, the implementation of case detection and medical mask usage should adapt to the condition of the infected compartment over time.

This section treats the control intervention as time-dependent variables, denoted as $u_{1}=u_{1}(t)$ and $u_{2}=u_{2}(t)$ . Consequently, the model in equations (2.1)–(2.5) now reads as:


(5.1) $$\begin{array}{rl} \frac{dS}{dt} & =\delta -(1-u_{2}(t)\xi )\beta SI_{1}-\mu S, \end{array}$$

(5.2) $$\begin{array}{rl} \frac{dE}{dt} & =p(1-u_{2}(t)\xi )\beta SI_{1}-(\theta +\epsilon +\mu )E, \end{array}$$

(5.3) $$\begin{array}{rl} \frac{dI_{1}}{dt} & =q(1-u_{2}(t)\xi )\beta SI_{1}+\theta E-(u_{1}(t)+k_{1}+d_{1}+\mu )I_{1}, \end{array}$$

(5.4) $$\begin{array}{rl} \frac{dI_{2}}{dt} & =\epsilon E+u_{1}(t)I_{1}-(\mu +d_{2}+k_{2})I_{2}, \end{array}$$

(5.5) $$\begin{array}{rl} \frac{dR}{dt} & =k_{1}I_{1}+k_{2}I_{2}-\mu R. \end{array}$$

We aim to minimize the cost function as follows:


(5.6) $$J=\int_{0}^{t_{f}}\left(\omega_{1}I_{1}+\omega_{2}I_{2}+\varphi_{1}u_{1}^{2}+\varphi_{2}u_{2}^{2}\right)\;dt,$$

where $\omega_{1}$ and $\omega_{2}$ are the weight parameters for the infected compartment, while $\varphi_{1}$ and $\varphi_{2}$ are the weight costs for the control variables. Each component on J can be described as follows:

— the cost owiing to all interventions, except the use of medical masks and case detection, in controlling the number of infected individuals $I_{1}$ and $I_{2}$ is described by $\int_{0}^{t_{f}}\left(\omega_{1}I_{1}+\omega_{2}I_{2}\right)\;dt;$

— the cost, due to the intensity of the intervention implemented, is given by $\int_{0}^{t_{f}}\left(\varphi_{1}u_{1}^{2}+\varphi_{2}u_{2}^{2}\right)\;dt.$

This optimal control construction aims to seek an optimal trajectory for $u_{1}^{*}$ and $u_{2}^{*}$ to minimize the cost function J . Mathematically, it is described by the following equation:


$$J(u_{1}^{*},u_{2}^{*})=\min_{\Theta }J(u_{1},u_{2}),$$

where $\Theta =\left\{(u_{1},u_{2})|u_{i}\;\;\text{is Lebesgue measurable function},u_{i}(t)\in [u_{i}^{\min},u_{i}^{\max}]\right\}$ is the set of admissible control. By applying the Pontryagin’s maximum principle, we define the Hamiltonian function as follows:


(5.7) $$H=\omega_{1}I_{1}+\omega_{2}I_{2}+\varphi_{1}u_{1}^{2}+\varphi_{2}u_{2}^{2}+\lambda_{1}\frac{dS}{dt}+\lambda_{2}\frac{dE}{dt}+\lambda_{3}\frac{dI_{1}}{dt}+\lambda_{4}\frac{dI_{2}}{dt}+\lambda_{5}\frac{dR}{dt}.$$

With this Hamiltonian function, we have the following Theorem 6.

**Theorem 6.** *Let the solution of the optimal control problem are* $S^{*}(t),E^{*}(t),I_{1}^{*}(t),I_{2}^{*}(t),R^{*}(t)$ *with it* $u_{1}^{*}(t)$ *and* $u_{2}^{*}(t)$ *. Then there exists an adjoint variable* $\lambda_{i}$ *for* i=1,2,3,4,5 *which satisfy the following system*:


(5.8) $$\frac{d\lambda_{1}}{dt}=p(1-u_{2}\xi )\beta I_{1}(\lambda_{1}-\lambda_{2})+q(1-u_{2}\xi )\beta I_{1}(\lambda_{1}-\lambda_{3})+\mu \lambda_{1},$$

(5.9) $$\begin{array}{rl} \frac{d\lambda_{2}}{dt} & =\theta (\lambda_{2}-\lambda_{3})+\epsilon (\lambda_{2}-\lambda_{4})+\mu \lambda_{2}, \end{array}$$

(5.10) $$\begin{array}{rl} \frac{d\lambda_{3}}{dt} & =-\omega_{1}+p(1-u_{2}\xi )\beta S(\lambda_{1}-\lambda_{2})+q(1-u_{2}\xi )\beta S(\lambda_{1}-\lambda_{3})\ldots \\ & +u_{1}(\lambda_{3}-\lambda_{4})+k_{1}(\lambda_{3}-\lambda_{5})+(\mu +d_{1})\lambda_{3}, \end{array}$$

(5.11) $$\frac{d\lambda_{4}}{dt}=-w_{2}+k_{2}(\lambda_{4}-\lambda_{5})+(\mu +d_{2})\lambda_{4},$$

(5.12) $$\begin{array}{rl} \frac{d\lambda_{5}}{dt} & =\mu \lambda_{5}, \end{array}$$

with their transversality conditions $\lambda_{i}(t_{f})=0$ for i=1,2,3,4,5 . The optimal trajectory on its admissible set is given by:


(5.13) $$u_{1}^{*}=\left\{\min\left\{\max\left\{u_{1}^{\min},\frac{I_{1}(\lambda_{3}-\lambda_{4})}{2\varphi_{1}}\right\}\right\},u_{1}^{\max}\right\},$$

(5.14) $$\begin{array}{rl} u_{2}^{*} & =\left\{\min\left\{\max\left\{u_{2}^{\min},\frac{\xi \beta SI_{1}(p\lambda_{2}+q\lambda_{3}-\lambda_{1})}{2\varphi_{2}}\right\}\right\},u_{2}^{\max}\right\}. \end{array}$$

For the derivation of Theorem 6, readers may refer to appendix G for the complete proof.

### 5.2. Numerical experiments of the optimal control problem

To solve the optimal control problem, we employed the forward–backward sweep method. Equations (5.1)–(5.5) was solved using forward sweep, a set of initial guesses for the control variables. Then, the adjoint system in equation (5.8) was solved backwards using the initial guess and the solution obtained from the previous step. Using the results, we computed the control variables as described in equation (5.13). All the steps were repeated until the convergence criteria were achieved. Further details and practical examples of the method can be found in Aldila *et al*. [8,43].

The numerical experiments in this section encompassed two distinct scenarios. The first scenario involved forecasting the incidence of cases in Indonesia, India, Lesotho and Angola for 30 years onwards up to 2050, with the optimal control variables incorporated into the model from 2021 to 2050. The second experiment focussed on simulating various scenarios for control implementation, including case detection only, using medical masks only and combining both interventions.

#### 5.2.1. Forecast of tuberculosis case incidence with time-dependent intervention

**Figure 6.**
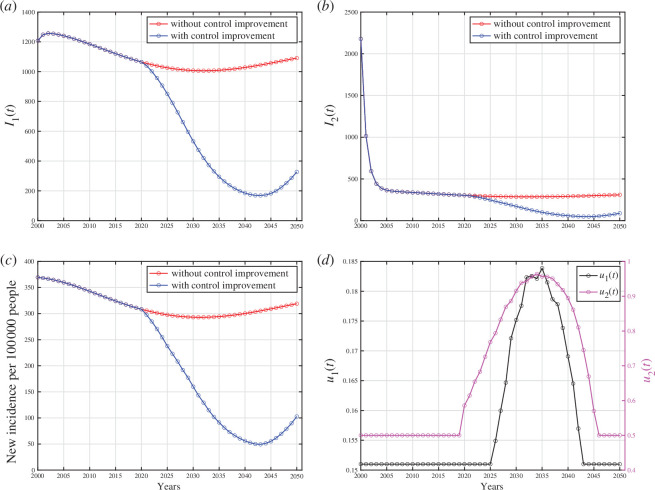
Forecasting and optimal control results for Indonesian data. Panels (*a*) and (*b*) represent the dynamic of $I_{1}$ and $I_{2}$ , respectively. Panel (*c*) represents the case incidence per 100 000 people without and with control improvement, while panel (*d*) shows the dynamic of control parameters.

The numerical experiments conducted in this section used the best-fit parameters in table 1. We forecasted the case incidence for each country until 2050 by implementing both case detection and the use of medical masks. The results for Indonesia are presented in figure 6. These forecasting results for India, Lesotho and Angola can be found in appendix H.

Based on the results in figure 6*a*,*b*, it is evident that the number of infected individuals continued to decrease even more significantly when control interventions were improved from 2021 until 2050, as control dynamics depicted in figure 6*d*. The control interventions from 2000 to 2020 used the data shown in table 1. This trend corresponds directly to the prediction of case incidence per 100 000 population in figure 6*c*, where it decreases significantly when control interventions improve but begins to rise when control interventions are reduced. It is worth highlighting that the substantial reduction shown in figure 6*c* starting from the year 2021 was owing to active case finding (corresponding to $u_{1}$ ) within the population. Mathematically, this sharp reduction was because of rapid transitions from the $I_{1}$ compartment to the $I_{2}$ compartment following the medical mask intervention. Without this $u_{1}$ , the number of detected cases was mainly contributed by the E compartment alone. Case detection should be improved from 2021, and it should start to decrease from 2035 onwards to minimize the intervention cost. Furthermore, we can see clearly that when the control interventions were gradually removed, then the number of cases began to rise again, highlighting their importance in controlling the outbreak. A similar pattern was observed in numerical simulation results for India, Lesotho and Angola, where the number of infected individuals decreased gradually with and without the control interventions, as shown in appendix H. Nevertheless, implementing control interventions speeds up the eradication process of the infected cases, compared with the case of no control intervention.

There are several important notes from numerical experiments for India, Lesotho and Angola. For simulation results using data from India, it is evident that interventions must be significantly enhanced in 2021 and start to decline in the year 2040. In an extreme scenario, the proportion of the population required to use medical masks reaches 100% from 2034 to 2040 and then begins to decrease significantly to 50%. Regarding data from Lesotho, no significant increase was observed in case detection interventions; it remained constant. Conversely, the intervention for medical mask usage needs to be significantly increased starting in 2021, then starting to decline in 2033. There is apparently no significant decrease in the new incidence rate in Lesotho with such control dynamics. Analysing data from Angola, it is evident that the number of people using medical masks must have significantly increased since 2021, even reaching 100% from that year onwards. This intervention starts to decline in the year 2041. On the other hand, the intervention for case detection must also have been increased since 2021. However, the difference lies in the fact that the intervention for case detection begins to decline in 2033 to offset the high intervention of medical mask usage, which undoubtedly incurs non-trivial costs.

#### 5.2.2. Assessing the effectiveness of various combinations of control strategies

The model incorporates two different control strategies: case detection and medical mask usage. As previously explained, case detection and medical mask interventions have different focuses. The case detection intervention aims to actively identify active TB cases in the field and provide them with appropriate treatment. On the other hand, the medical mask intervention is more oriented towards preventing the spread of TB by encouraging infected individuals to protect the population by using medical masks. In essence, case detection is a mitigation intervention, while medical mask use is a preventative measure.

The effectiveness of various combinations of these control strategies was investigated in this section. In particular, the following three scenarios were considered in our numerical simulations:

– implementation of both case detection and medical mask usage, i.e. $u_{1}(t)\in [0.151,1]$ and $u_{2}(t)[0.5,1]$ ;
– implementation of case detection only, i.e. $u_{1}(t)\in [0.151,1]$ and $u_{2}(t)=0.5$ ; and
– implementation of medical mask usage only, i.e. $u_{1}(t)=0.151$ and $u_{2}(t)\in [0.5,1]$ .

**Figure 7.**
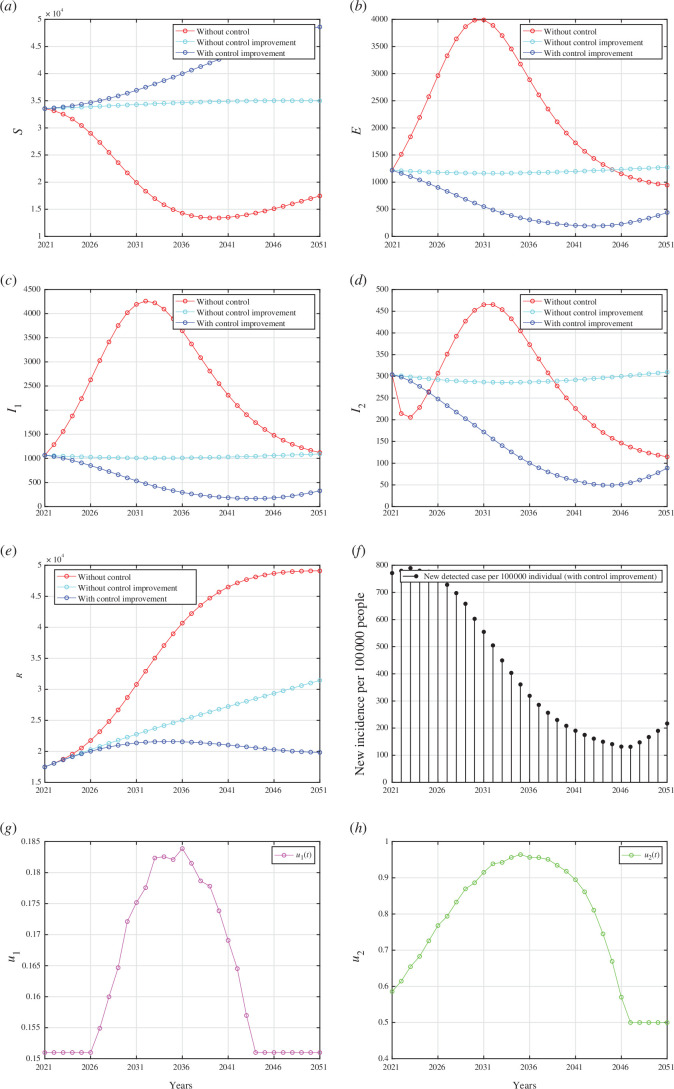
Forecasting and optimal control results for Indonesian data from 2021 to 2051 when case detection and medical mask use are implemented together. Panels (*a*) to (*e*) represent the dynamic of $S,E,I_{1},I_{2}$ and R , respectively. Panel (*f*) represents the case incidence per 100 000 people while panels (*g*) and (*h*) show the dynamic of control $u_{1}$ and $u_{2}$ , respectively.

**Figure 8.**
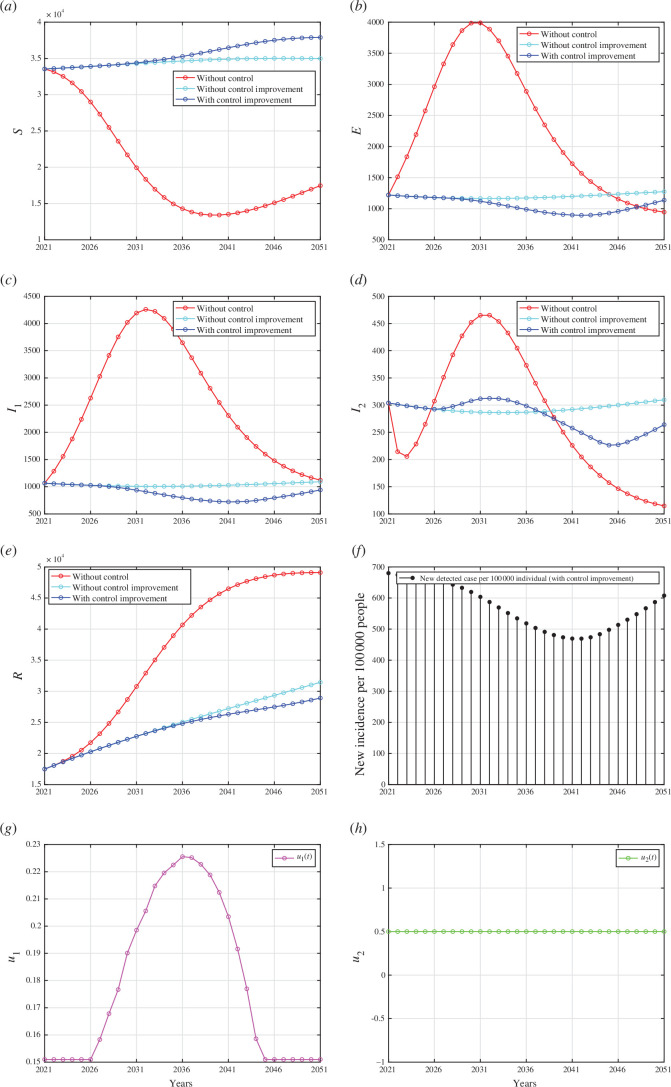
Forecasting and optimal control results for Indonesian data from 2021 to 2051 when case detection intervention improved, but medical mask left as a constant at $u_{2}=0.5$ . Panels (*a*) to (*e*) represent the dynamic of $S,E,I_{1},I_{2}$ and R , respectively. Panel (*f*) represent the case incidence per 100 000 people while panels (*g*) and (*h*) show the dynamic of control $u_{1}$ and $u_{2}$ , respectively.

**Figure 9.**
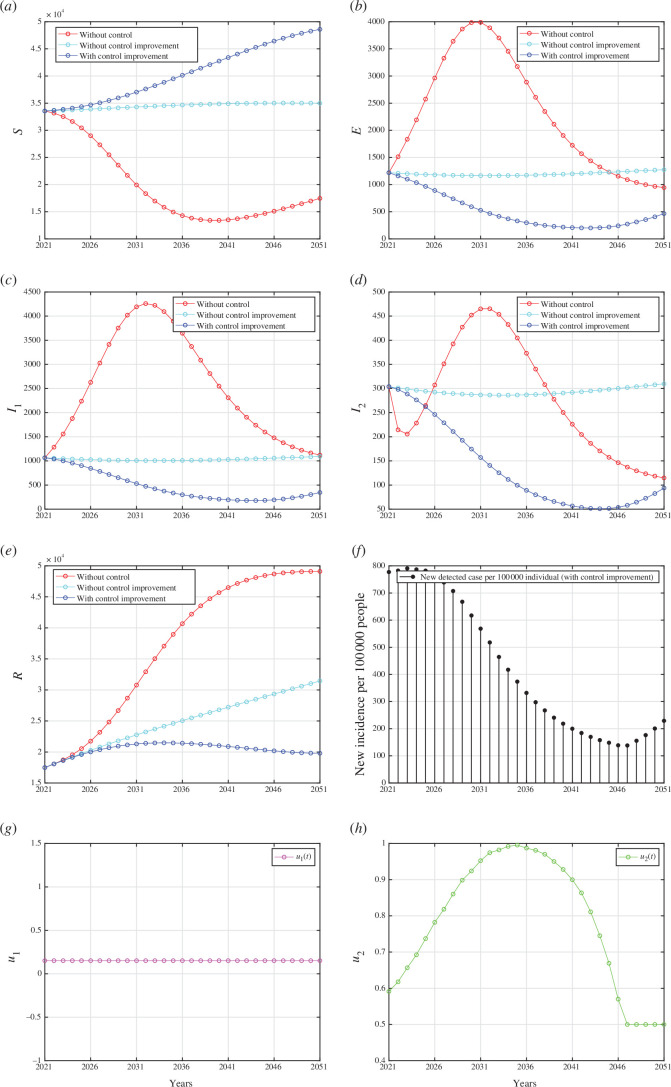
Forecasting and optimal control results for Indonesian data from 2021 to 2070 when medical mask intervention improved, but case detection left as a constant at $u_{1}=0.151$ . Panels (*a*) to (*e*) represent the dynamic of $S,E,I_{1},I_{2}$ and R , respectively. Panel (*f*) represent the case incidence per 100 000 people while panels (*g*) and (*h*) show the dynamic of control $u_{1}$ and $u_{2}$ , respectively.

The lower bounds of each control parameter are derived from the best-fit parameters outlined in table 1, whereas the upper bounds are set equal to 1. All numerical results are given in appendix I. In each panel depicting the dynamics of every compartment from figures 7–9 (panel (a) to (e)), we assess three distinct scenarios: first, when no controls were implemented $\left(u_{1}=u_{2}=0\right)$ ; second, when interventions regarding case detection and the use of medical masks remained constant until the year 2051; and third, when improvements were made in case detection and/or medical mask utilization.

##### 5.2.2.1. Scenario 1: improvement in case detection and medical mask use

The results of the first scenario are presented in appendix I, where both case detection and medical mask interventions were simultaneously implemented. Appendix I depicts the dynamics of compartments $S,E,I_{1},I_{2}$ and R , respectively. It is evident that by improving both case detection and medical mask use interventions, as shown in appendix I, the number of healthy individuals can be significantly increased, and the number of infected individuals can be significantly reduced. As a result, it can be observed that the number of detected infected individuals steadily decreased in proportion to the decrease in the number of infected individuals (see appendix I). However, as the intervention diminishes from 2036 onwards, the incidence of new cases rises, particularly evident from 2046. Unlike the intervention of medical masks, which has required improvement since 2021, the intervention of case detection may remain constant at $u_{1}=0.151$ from 2021 until 2026, subsequently increasing until 2036. Under this scenario, the total number of averted infections reaches $1.226\times 10^{5}$ .

##### 5.2.2.2. Scenario 2: improvement in case detection only

In this scenario, we conducted numerical simulations to understand the effects of case detection as a single intervention that we need to improve in controlling TB transmission in Indonesia. By contrast, the intervention of medical mask use remains constant at $u_{2}=0.5$ . The results are presented in appendix I. The case detection rate in this scenario is illustrated in appendix I, which is significantly higher compared with the previous scenario where case detection intervention was accompanied by medical mask usage in TB mitigation efforts (see appendix I). Despite the intensive implementation of case detection intervention, the reduction in the number of infected individuals was not as effective as in the previous scenario (see appendix I). This scenario’s total number of infections averted was $8.9\times 10^{4}$ .

##### 5.2.2.3. Scenario 3: improvement in medical mask use only

For the last scenario, we examined the impact of the improvement on medical mask use in preventing the spread of TB while keeping the case detection constant at $u_{1}=0.151$ . The results of the numerical simulation are shown in appendix I. Since the use of medical masks was the sole intervention in this scenario, the intervention intensity was higher compared with the first scenario, as indicated in appendix I. We can see that the intervention of medical masks alone reduced the total number of infected cases better than the intervention of case detection alone. This is supported by the fact that this scenario’s total number of infections averted was $1.295\times 10^{4}$ . This was smaller than the first two scenarios.

##### 5.2.2.4. Cost-effectiveness analysis

Based on the numerical simulations conducted for all three scenarios mentioned above, implementing both interventions (scenario 3) yields the most significant results in the number of infections prevented, but it is only slightly different from scenario 1, where both interventions improved. However, this may come at a higher intervention cost. Therefore, an analysis was needed to assess the effectiveness of these strategies relative to the costs incurred. To do so, we used the average cost-effectiveness ratio (ACER) to compare the effectiveness of our three scenarios. The formula to calculate ACER is given by


(5.15) $$\text{ACER}=\frac{\text{total cost for intervention (TC)}}{\text{total number of infections averted (TIA)}}.$$

The formula for ACER is given by equation (5.15), where TC is the total cost of interventions $u_{1}$ and $u_{2}$ , while TIA is the total number of infections averted from compartments E , $I_{1}$ and $I_{2}$ . A smaller value of ACER represents a more cost-effective strategy.

Table 2 shows that ACER for scenario 1 was the smallest, followed by scenarios 2 and 3. Our results demonstrated that combining both interventions provided better cost-effectiveness than the other two scenarios.

**Table 2.** The cost-effectiveness analysis (ACER) for all three scenarios.

scenario	TC	TIA	ACER
1	$1.281\times 10^{4}$	$1.226\times 10^{5}$	0.1044
2	$1.878\times 10^{4}$	$8.9\times 10^{4}$	0.211
3	$1.295\times 10^{4}$	$1.227\times 10^{5}$	0.1055

The next cost-effectiveness analysis is the infection averted ratio (IAR) analysis. This analysis assessed the effectiveness of interventions to prevent the spread of infectious diseases. It quantifies the impact of an intervention by comparing the number of infections averted owing to the intervention to the total number of recovered individuals owing to the implementation of control programmes. Hence, the formula to calculate IAR is given by:


(5.16) $$\text{IAR}=\frac{\text{total number of infections averted (TIA)}}{\text{total recovered (TR)}}.$$

A higher IAR value indicates that the intervention is more efficient in preventing infections relative to the recovered individual. The IAR analysis helps decision-makers prioritize interventions and allocate resources effectively to maximize the impact of disease prevention efforts. Using this formula, we calculate the IAR for each scenario and yield that the IAR for scenario 1 is 0.199, scenario 2 is 0.1228 and scenario 3 is 0.2. Hence, we find that the intervention of medical mask use alone is the best strategy using the IAR indicator, followed by the combination of medical mask and case detection, and finally, medical mask use alone.

From the above cost-effectiveness analysis and the GSA, we can see that implementing medical masks is more successful in reducing the number of infected individuals than case detection. However, we need to be careful that there are several reasons why medical masks may be considered a better strategy for TB control compared with case detection in certain situations. First, a medical mask is a preventive measure by reducing the probability of successful TB transmission. Medical mask use can help prevent the spread of TB in crowded or poorly ventilated settings, such as healthcare facilities, prisons and shelters, where close contact between individuals increases the risk of TB transmission. By contrast, case detection primarily targets identifying and treating individuals already infected with TB, which may not effectively prevent transmission from undetected cases. Second, medical masks are relatively more universal in their applications and more efficient in the cost of implementation. In resource-limited settings where healthcare infrastructure is limited, medical masks may offer a cost-effective approach to TB control. It is important to note that while medical masks can play a valuable role in TB control, they are not a standalone solution and should be integrated into comprehensive TB control programs alongside other interventions, including case detection, treatment, infection control measures, and public health education.

## 6. Discussion and conclusion

A TB model that includes the effects of medical mask use and active case finding is proposed in this article. This nonlinear system of ODEs consists of five variables representing various classes of the human population and 14 parameters. The analysis of the mathematical model concerning the threshold reproduction number, $R_{c}$ , demonstrates that implementing medical mask usage and active case finding can potentially mitigate the spread of TB effectively. From model equilibrium analysis, we find that our model always exhibits a transcritical bifurcation at $R_{c}=1$ . This finding suggests that the persistence of TB occurs when the reproductive number, $R_{c}>1$ , while TB will become extinct if $R_{c}<1$ (see appendix F).

Our model is parameterized using yearly incidence data on TB cases per 100 000 population from four different countries: Indonesia, India, Lesotho and Angola (see §2.2). This parameterization revealed that the reproduction numbers for all four countries consistently exceeded 1, suggesting the potential for sustained TB endemicity. Nevertheless, our model’s forecasting results indicated a declining trend in TB cases within these countries in the coming years (refer to figure 6; appendices H).

Numerical experiments have been carried out to provide evidence that medical masks and proactive case identification can significantly enhance the potential of eliminating TB from the population. As the parameter ξ indicated within our model, a better quality of medical masks also contributed to the reduction of $R_{c}$ . In addition, as the probability of fast disease progression, q increased, a more intense use of medical masks and proactive efforts by the government to conduct active case finding became imperative for the effective eradication of TB. Our sensitivity analysis showed that undetected and detected active TB dynamics were highly responsive to active case findings. This sensitivity was most pronounced at the outset of the intervention and gradually diminished over time.

Using this parameterization, we further developed our model into an optimal control model to analyse the effectiveness of case detection and usage of medical masks as a function of time in controlling TB. From the results obtained, it is evident that the combination of both case detection and the usage of medical masks proved to be a cost-effective and effective strategy for mitigating the spread of TB, notably in reducing the incidence of infection. These findings can provide valuable insights into the complexity of TB transmission in these four countries. The differing focus of interventions can be considered in the future and adapted to the on-ground conditions.

Our research shows a big potential for medical masks to be used as a non-pharmaceutical intervention for TB eradication programmes (even with a small efficacy of only 56% [34]). Implementing medical masks as a TB intervention over a 50 year eradication programme presents several challenges and considerations. While medical masks may offer some degree of protection against TB transmission, their long-term feasibility and effectiveness in such a programme would depend on various factors such as acceptance and compliance, access and affordability and cultural and social factors. Implementing medical masks for such a long period may require significant efforts to promote awareness, education and behaviour change. Ensuring consistent compliance with mask-wearing guidelines over many years could be challenging, especially in regions with low TB prevalence or where perceptions of risk fluctuate. Furthermore, governments and health authorities would need to invest in infrastructure, supply chains and subsidies to make medical masks accessible to all socio-economic groups over the long term. In summary, while implementing medical masks as a TB intervention over a 50 year eradication programme is theoretically feasible, it would require comprehensive planning, sustained investment, community engagement and integration with broader TB control strategies. Although it is a challenging effort to implement this on a big scale, like on a country scale, the implementation of medical mask usage can begin at the grassroots level, starting with the smallest social circles such as households with TB-infected individuals, hospitals [56] and similar environments.

As mentioned earlier, the challenge in TB control globally persists owing to several factors. One major issue is the emergence of drug-resistant TB strains, such as MDR-TB and extensively drug-resistant TB, which are harder and costlier to treat. Additionally, TB often affects marginalized and vulnerable populations, making access to healthcare and proper diagnosis a challenge. Addressing these challenges requires a multi-pronged approach, including augmented financial resources, enhanced diagnostic capabilities and strengthened healthcare systems to ensure equitable access to TB care. Hence, it is important to continue our efforts to refine the model by integrating the factors above into our new model for future studies.

## Figures and Tables

**Figure 10.**
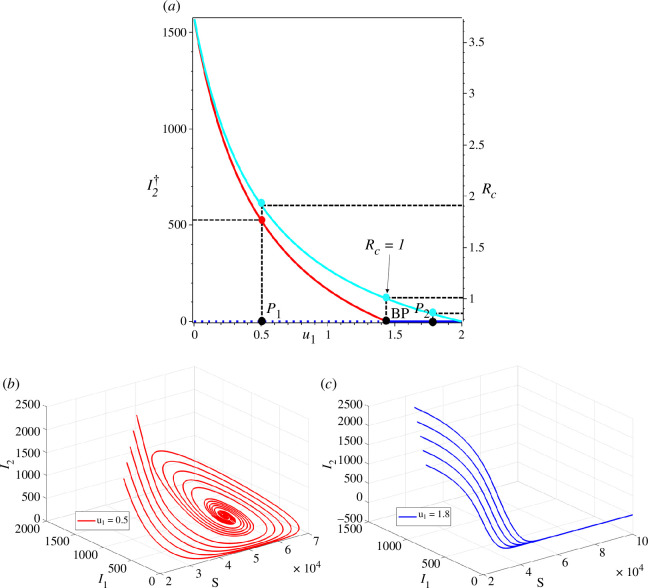
(*a*) Bifurcation diagram of equations (2.1)–(2.5) represented by $I_{1}$ and $R_{c}$ with respect to $u_{1}$ . The solid lines represent the stable branch of the equilibrium point and the dashed line represents the unstable branch of the equilibrium point. Red, blue and cyan curves represent the endemic equilibrium, TB-free equilibrium and $R_{c}$ , respectively. Panels (*b*) and (*c*) show the trajectories in $S-I_{1}-I_{2}$ phase planes at two different sample points $P_{1}(u_{1}=0.5)$ and $P_{2}(u_{1}=1.8)$ , respectively. All solutions were obtained numerically using various initial conditions.

**Figure 11.**
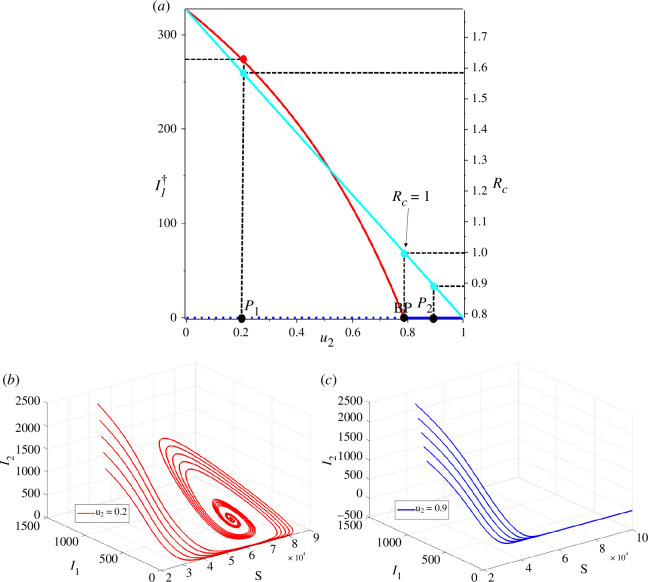
(*a*) Bifurcation diagram of system equations (2.1)–(2.5) represented by $I_{1}$ and $R_{c}$ with respect to $u_{2}$ . The solid lines represent the stable branch of the equilibrium point and the dotted line represents the unstable branch of the equilibrium point. The red and cyan curves represent the endemic equilibrium and the $R_{c}$ , respectively. Panels (*b*) and (*c*) show trajectories solution of $S,I_{1}$ and $I_{2}$ at two different sample points $P_{1}(u_{2}=0.2)$ and $P_{2}(u_{2}=0.9)$ . All solutions were obtained numerically using various initial conditions.

**Figure 12.**
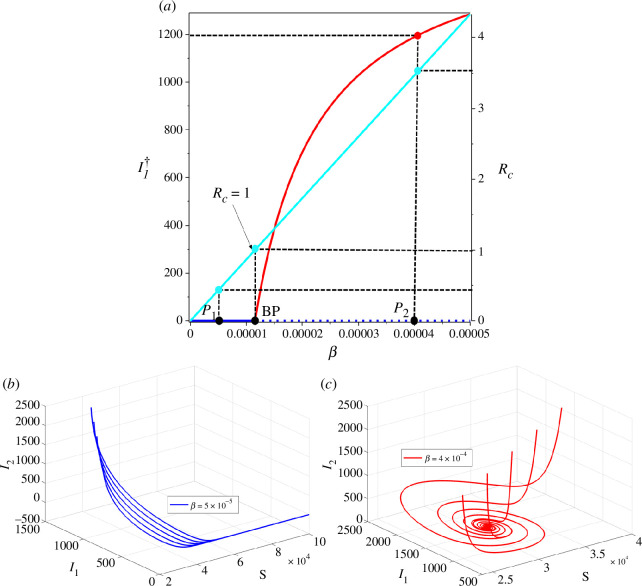
(*a*) Bifurcation diagram of equations (2.1)–(2.5) represented by $I_{1}$ and $R_{c}$ with respect to β . The red and cyan curves represent the endemic equilibrium and the $R_{c}$ , respectively. Panels (*b*) and (*c*) show the trajectories of the solution for two different sample points $P_{1}(\beta =0.000005)$ and $P_{6}(\beta =0.00004)$ . All solutions were obtained numerically using various initial conditions.

**Figure 13.**
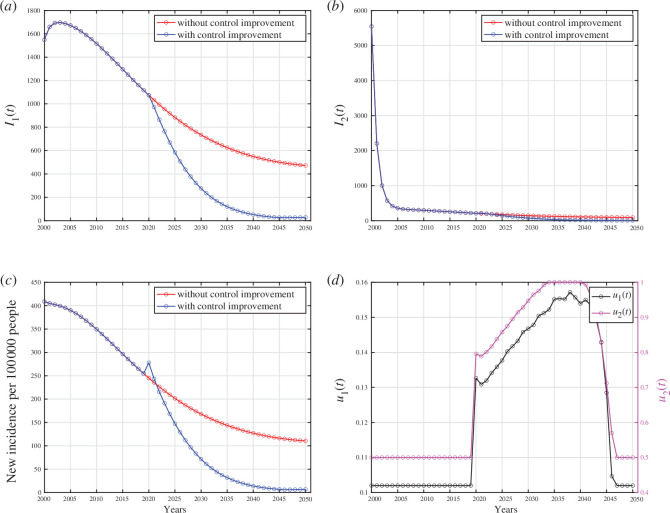
Forecasting and optimal control results for India data. Panels (*a*) and (*b*) represent the dynamic of $I_{1}$ and $I_{2}$ , respectively. Panel (*c*) represents the case incidence per 100 000 people with and without control, while panel (*d*) shows the dynamic of controls.

**Figure 14.**
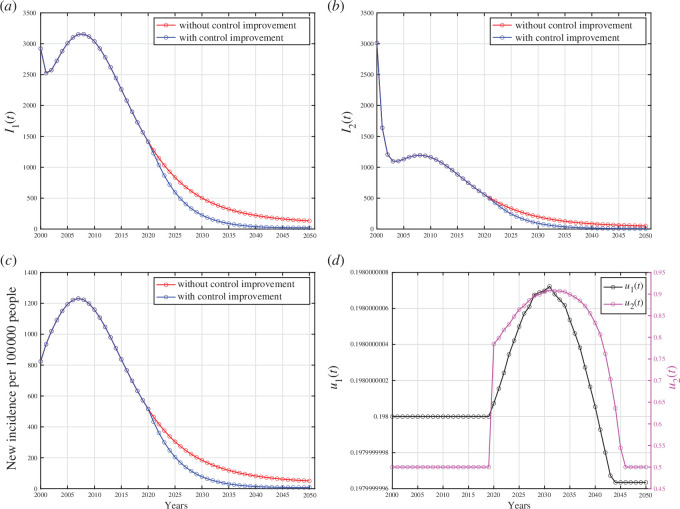
Forecasting and optimal control results for Lesotho data. Panels (*a*) and (*b*) represent the dynamic of $I_{1}$ and $I_{2}$ , respectively. Panel (*c*) represents the case incidence per 100 000 people with and without control, while panel (*d*) shows the dynamic of controls.

**Figure 15.**
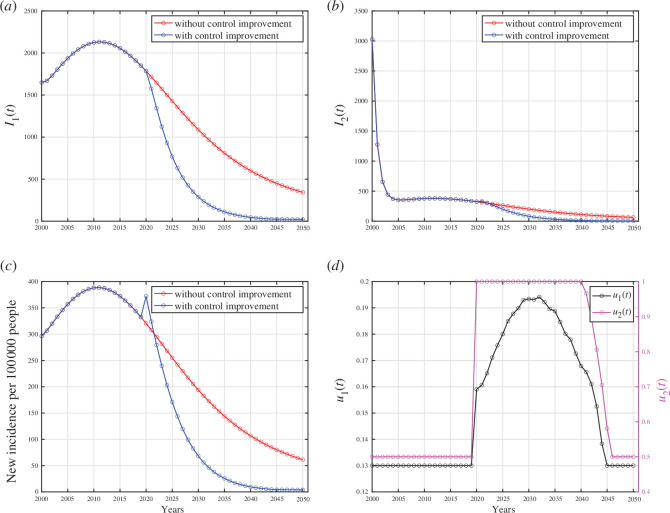
Forecasting and optimal control results for Angola data. Panels (*a*) and (*b*) represent the dynamic of $I_{1}$ and $I_{2}$ , respectively. Panel (*c*) represents the case incidence per 100 000 people with and without control, while panel (*d*) shows the dynamic of controls.

## Data Availability

The data used in this research originates from The World Bank Open Data (https://data.worldbank.org/indicator/SH.TBS.INCD). This website provides various types of data, including the incidence of tuberculosis (per 100 000 people) from around the world, which can be found at the following link: [57]. The World Bank Group makes data publicly available in accordance with open data standards and licenses datasets under the 'Creative Commons Attribution 4.0 International license (CC-BY 4.0)'. Please refer to the following link for the source of this statement: https://datacatalog.worldbank.org/public-licenses#cc-by. With this license type, it allows any users (including myself) to copy, modify and distribute the data in any format for any purpose, including commercial use. Users are only obligated to give appropriate credit (attribution) and indicate if they have made any changes, including translations. The complete incidence data can be downloaded directly from The World Bank Group's website using the following URL: https://api.worldbank.org/v2/en/indicator/SH.TBS.INCD?downloadformat=excel. For the data used in this research, we only extracted data for four countries: Indonesia, India, Lesotho and Angola. This set of data for these four countries can be accessed as an Excel file through the following link: [58] . We have also uploaded our MATLAB code and all figures to the same link.

## Appendix A. Proof of invariant region Ω

The invariant region of the proposed model can be shown below, following the approach in [59,60]. The solutions with non-negative initial conditions remain in a neighbourhood of the closed positive hyperspace, $\Omega \in R_{+}^{5}$ , for t≥0 , that is, $(S,E,I_{1},I_{2},R)\in {\bar{R}}_{+}^{5}$ , for

$$\begin{array}{ll} & S^{\prime }{|}_{S=0}=\delta \geq 0,\\ & E_{c}^{\prime }{|}_{E_{c}=0}=p(1-u_{2}\xi )\beta SI_{1}\geq 0,\\ & I_{1}^{\prime }{|}_{I_{1}=0}=\theta E\geq 0,\\ & I_{2}^{\prime }{|}_{I_{2}=0}=\epsilon E+u_{1}I_{1}\geq 0,\\ & R^{\prime }{|}_{R=0}=k_{1}I_{1}+k_{2}I_{2}\geq 0. \end{array}$$

It can be seen that the gradient on the boundary of $R_{+}^{5}$ is always positive. Hence, the solution will always be non-negative for all t>0 . Summing all right-hand side of equations (2.1)–(2.5), we have:

$$\frac{dN}{dt}=\delta -\mu N-d_{1}I_{1}-d_{2}I_{2}\leq \delta -\mu N.$$

Hence, using the integrating factor method, the solution of N satisfying:

$$N(t)\leq \frac{\delta }{\mu }+\left(N(0)-\frac{\delta }{\mu }\right)e^{\mu t}.$$

It can be seen that if $N(0)<\frac{\delta }{\mu }$ , then N(t) will monotonically increasing and tends to $\frac{\delta }{\mu }$ . If $N(0)>\frac{\delta }{\mu }$ , then N(t) will be monotonically decreasing and tends to $\frac{\delta }{\mu }$ . If $N(0)=\frac{\delta }{\mu }$ , then N(t) always stays inside of Ω . Therefore, all feasible solutions of the modelequations (2.1)–(2.5) enter the region Ω implying that the region is an attracting set. Hence the proof is complete.

## Appendix B. Proof of Theorem 1: derivation of the control reproduction number

Using the next generation matrix (NGM) method [61], here are steps to find $R_{c}$ :

– create the Jacobian matrix J of the infected compartment by linearization around the DFE point as follows.

$$\begin{array}{r} \text{J}=\left[\begin{array}{ccc} -(\theta +\epsilon +\mu ) & \frac{p\beta \delta (1-u_{2}\xi )}{\mu } & 0\\ 0 & \frac{q\beta \delta (1-u_{2}\xi )}{\mu }-(u_{1}+k_{1}+d_{1}+\mu ) & 0\\ \epsilon & u_{1} & -(\mu +d_{2}+k_{2}) \end{array}\right]; \end{array}$$

– decompose the Jacobian matrix as J=T+Σ with T is the transmission matrix and Σ is the transition matrix as follows.

$$\begin{array}{rlr} \text{T} & =\left[\begin{array}{ccc} 0 & \frac{p\beta \delta (1-u_{2}\xi )}{\mu } & 0\\ 0 & \frac{q\beta \delta (1-u_{2}\xi )}{\mu } & 0\\ 0 & 0 & 0 \end{array}\right]\text{and}\;\Sigma & =\left[\begin{array}{ccc} -(\theta +\epsilon +\mu ) & 0 & 0\\ \phi & -(u_{1}+k_{1}+d_{1}+\mu ) & 0\\ \epsilon & u_{1} & -(\mu +d_{2}+k_{2}) \end{array}\right]; \end{array}$$

– as we can see, the T matrix consists of one row that is entirely zeros, then we must construct the E matrix, which columns are unit vectors that correspond with a non-zero row of T.

$$\begin{array}{r} \text{E}=\left[\begin{array}{cc} 1 & 0\\ 0 & 1\\ 0 & 0 \end{array}\right]\text{and}\;{\text{E}}^{T}=\left[\begin{array}{ccc} 1 & 0 & 0\\ 0 & 1 & 0 \end{array}\right]; \end{array}$$

– use one of NGM’s formulas that is denoted by the K matrix as follows.

$$\begin{array}{r} \text{K}=\left[\begin{array}{cc} \frac{p\beta \delta \theta (1-u_{2}\xi )}{\mu (\theta +\epsilon +\mu )(u_{1}+k_{1}+d_{1}+\mu )} & \frac{p\beta \delta (1-u_{2}\xi )}{\mu (u_{1}+k_{1}+d_{1}+\mu )}\\ \frac{q\beta \delta \theta (1-u_{2}\xi )}{\mu (\theta +\epsilon +\mu )(u_{1}+k_{1}+d_{1}+\mu )} & \frac{q\beta \delta (1-u_{2}\xi )}{\mu (u_{1}+k_{1}+d_{1}+\mu )} \end{array}\right]; \end{array}$$

– from the K matrix, we get the determinant of K is zero. Therefore, we use another one of NGM’s formulas involving a small domain. Hence, $R_{c}$ is given by:

$$\begin{array}{r} R_{c}=\frac{\delta \beta (1-u_{2}\xi )(p\theta +q\theta +q\epsilon +q\mu )}{\mu (\theta +\epsilon +\mu )(u_{1}+k_{1}+d_{1}+\mu )}. \end{array}$$

## Appendix C. Proof of Theorem 2: local stability criteria of $E_{1}$

We use Van den Driessche and Watmough’s approach [45] to analyse the local stability of DFE. First, define $\text{x}=(x_{1},x_{2},x_{3},x_{4},x_{5}{)}^{T}$ with $x_{1},x_{2},x_{3},x_{4}\;and\;x_{5}$ is the number of individuals in each compartment $E,I_{1},I_{2},S$ and R , respectively. Then, define ${\text{X}}_{s}$ as the set of DFE populations:

$$\begin{array}{r} {\text{X}}_{s}=\{\left(x_{1}=0,x_{2}=0,x_{3}=0,x_{4}=\frac{\delta }{\mu },x_{5}=0\right)\}. \end{array}$$

Define $f_{i}(x)$ for i=1,2,…,5 which represents $\frac{dE}{dt},\frac{dI_{1}}{dt},\frac{dI_{2}}{dt},\frac{ds}{dt}$ and $\frac{dR}{dt}.$ Note that the initial condition for each population must have a non-negative value following this condition:

$$\begin{array}{r} {\dot{x}}_{i}=f_{i}(\text{x})=F_{i}(\text{x})-V_{i}(\text{x}),i=1,2,3,4,5. \end{array}$$

Then, we have

(C 1)
$$\begin{array}{r} F(\text{x})=\left[\begin{array}{c} p(1-u_{2}\xi )\beta SI_{1}\\ q(1-u_{2}\xi )\beta SI_{1}\\ 0\\ 0\\ 0 \end{array}\right] \end{array}$$

and

(C 2)
$$\begin{array}{r} V(\text{x})=\left[\begin{array}{c} (\theta +\epsilon +\mu )E\\ (u_{1}+k_{1}+d_{1}+\mu )I_{1}-\theta E\\ (\mu +d_{2}+k_{2})I_{2}-\epsilon E-u_{1}I_{1}\\ (1-u_{2}\xi )\beta SI_{1}+\mu S-\delta \\ \mu R-k_{1}I_{1}-k_{2}I_{2} \end{array}\right], \end{array}$$

where $V(\text{x})=V^{-}(\text{x})-V^{+}(\text{x})$ are given by

(C 3)
$$\begin{array}{r} V^{+}(\text{x})=\left[\begin{array}{c} 0\\ 0\\ \epsilon E+u_{1}I_{1}\\ \delta \\ k_{1}I_{1}+k_{2}I_{2} \end{array}\right] \end{array}$$

and

(C 4)
$$\begin{array}{r} V^{-}(\text{x})=\left[\begin{array}{c} (\theta +\epsilon +\mu )E\\ (u_{1}+k_{1}+d_{1}+\mu )I_{1}\\ (\mu +d_{2}+k_{2})I_{2}\\ (1-u_{2}\xi )\beta SI_{1}+\mu S\\ \mu R \end{array}\right]. \end{array}$$

After that, analyse whether DFE is locally asymptotically stable when $R_{c}<1$ using five axioms from Van den Driessche and Watmough:

— if $x_{i}\geq 0$ , then $F_{i},V_{i}^{+},V_{i}^{-}\geq 0$ for i=1,2,…,5. All parameters and variables have non-negative input. Based on that, the first axiom is satisfied;
— if $x_{i}=0$ , then $V^{-}=0$ for i=1,2,3. By substituting $x_{i}=0$ for i=1,2,3 to equation (3.5), we will get $V_{i}^{-}=\text{0}$ . Hence, the second axiom is satisfied;
— $F_{i}=0$ if i>3 . For i=4,5 , as we can see from equation (3.2) that $F_{4}=F_{5}=0$ , then the third axiom is satisfied; and
— if $x_{i}\in {\text{X}}_{s}$ , then $F_{i}=\text{0}$ and $V_{i}^{+}=\text{0}$ for i=1,2,3. Substitute $x_{i}=0$ to equations (3.2) and (5.8), then we will get $F_{i}=\text{0}$ and $V_{i}^{+}=\text{0}$ for i=1,2,3 . Hence, the fourth axiom is satisfied.

If $F_{i}=\text{0}$ , then all eigenvalues of $D\text{\{f\}}(x_{0})$ have a negative real part. By substituting $F_{i}(\text{x) = \{0\}}$ to $f_{i}(x)$ , we obtain:

$$\begin{array}{ll} f_{1}:=\frac{dE}{dt} & =-(\theta +\epsilon +\mu )E,\\ f_{2}:=\frac{dI_{1}}{dt} & =\theta E-(u_{1}+k_{1}+d_{1}+\mu )I_{1},\\ f_{3}:=\frac{dI_{2}}{dt} & =\epsilon E+u_{1}I_{1}-(\mu +d_{2}+k_{2})I_{2},\\ f_{4}:=\frac{dS}{dt} & =\delta -(1-u_{2}\xi )\beta SI_{1}-\mu S,\\ f_{5}:=\frac{dR}{dt} & =k_{1}I_{1}+k_{2}I_{2}-\mu R. \end{array}$$

Then, we obtain the Jacobian matrix of $f_{i}(x)$ that is evaluated at DFE as follows:

$$\begin{array}{r} D\text{f}(x_{0})=\left[\begin{array}{ccccc} -(\theta +\epsilon +\mu ) & 0 & 0 & 0 & 0\\ 0 & -(u_{1}+k_{1}+d_{1}+\mu ) & 0 & 0 & 0\\ \epsilon & u_{1} & -(\mu +d_{2}+k_{2}) & 0 & 0\\ 0 & -\frac{\delta \beta (1-u_{2}\xi )}{\mu } & 0 & -\mu & 0\\ 0 & k_{1} & k_{2} & 0 & -\mu \end{array}\right], \end{array}$$

with eigenvalues of the matrix above are $-(\theta +\epsilon +\mu ),-(u_{1}+k_{1}+d_{1}+\mu ),-(\mu +d_{2}+k_{2}),-\mu$ and −μ. Note that all parameters have non-negative values, it makes all eigenvalues have a negative real part. Then, we can conclude that the last axiom is satisfied too.

According to these results, all axioms are satisfied. Furthermore, we can guarantee in the second theorem that the disease-free equilibrium of system of equation (1) is locally asymptotically stable.

## Appendix D. Proof of Theorem 3: global stability of the disease-free equilibrium

To prove that the DFE is GAS whenever $R_{0}<1$ , we have to verify the conditions $C_{1}$ to $C_{3}.$ Using the result by Van den Driessche & Watmough [45], we obtain that the DFE $E_{1}$ is Locally Asymptotically Stable (LAS) when $R_{0}<1$ , so the condition $C_{1}$ is verified. Next, we re-write the model (equations (2.1)–(2.5)) in the form given in equation 3.3 as:

(D 1)
$$\frac{dX}{dt}=F(X,I)=\left(\begin{array}{c} \delta -(1-u_{2}\xi )\beta SI_{1}-\mu S\\ k_{1}I_{1}+k_{2}I_{2}-\mu R \end{array}\right)$$

and

(D 2)
$$\frac{dI}{dt}=G(X,I)=\left(\begin{array}{c} p(1-u_{2}\xi )\beta SI_{1}-(\theta +\epsilon +\mu )E\\ q(1-u_{2}\xi )\beta SI_{1}+\theta E-(u_{1}+k_{1}+d_{1}+\mu )I_{1}\\ \epsilon E+u_{1}I_{1}-(\mu +d_{2}+k_{2})I_{2} \end{array}\right).$$

We have that

(D 3)
$$\frac{dX}{dt}=F(X_{0},0)\Leftrightarrow \left\{ \begin{array}{l} S^{\prime }=\delta -\mu S,\\ R^{\prime }=-\mu R. \end{array}\right.$$

Equation (D3) has a unique equilibrium point $(\frac{\delta }{\mu },0,0,0,0)$ which is globally asymptotically stable. Hence, the condition $C_{2}$ is satisfied. Linearizing equations (D1) and (D2) yields the Metzler matrix ( $A=D_{Z}G(E_{1})$ ) given as:

$$A=\left(\begin{array}{ccccc} -\mu & 0 & -(1-u_{2}\xi )\beta S^{0} & 0 & 0\\ 0 & -(\theta +\epsilon +\mu ) & p(1-u_{2}\xi )\beta S^{0} & 0 & 0\\ 0 & \theta & q(1-u_{2}\xi )\beta S^{0}-(u_{1}+k_{1}+d_{1}+\mu ) & 0 & 0\\ 0 & \epsilon & u_{1} & -(\mu +d_{2}+k_{2}) & 0\\ 0 & 0 & k_{1} & k_{2} & -\mu \end{array}\right).$$

Computing $\hat{G}(X,Z)$ and after some algebraic manipulation, we have that

$$\hat{G}(X,I)=AI-G(X,I)=\left(\begin{array}{c} \delta +(1-u_{2}\xi )\beta I_{1}(S^{0}-S)\\ p(1-u_{2}\xi )\beta I_{1}(S^{0}-S)\\ q(1-u_{2}\xi )\beta I_{1}(S^{0}-S)\\ 0\\ 0\\ 0 \end{array}\right).$$

Thus, $(S^{0}-S)=S(\frac{S^{0}}{S}-1)$ is positive owing to our assumption, and hence $\hat{G}(X,I)\geq 0$ . The condition $C_{3}$ is also satisfied. We can conclude that our model is globally asymptotically stable.

## Appendix E. Proof of Theorem 5: non-existence of backward bifurcation

We use Castillo–Cahvez & Song theorem [47] to analyse the stability of endemic equilibrium from equations (2.1)–(2.5). First, suppose each compartment is as follows:

$$\begin{array}{r} S=x_{1},E=x_{2},I_{1}=x_{3},I_{2}=x_{4},R=x_{5}. \end{array}$$

Then, redefine the system as:

(E 1)
$$\begin{array}{l} g_{1}:=\frac{dx_{1}}{dt}=\delta -(1-u_{2}\xi )\beta x_{1}x_{3}-\mu x_{1},\\ g_{2}:=\frac{dx_{2}}{dt}=p(1-u_{2}\xi )\beta x_{1}x_{3}-(\theta +\epsilon +\mu )x_{2},\\ g_{3}:=\frac{dx_{3}}{dt}=q(1-u_{2}\xi )\beta x_{1}x_{3}+\theta x_{2}-(u_{1}+k_{1}+d_{1}+\theta )x_{3},\\ g_{4}:=\frac{dx_{4}}{dt}=\epsilon x_{2}+u_{1}x_{3}-(\mu +d_{2}+k_{2})x_{4},\\ g_{5}:=\frac{dx_{5}}{dt}=k_{1}x_{3}+K_{2}x_{4}-\mu x_{5}. \end{array}$$

Choose β as the bifurcation parameter, then setting $R_{c}=1$ and we obtain:

(E 2)
$$\begin{array}{r} \beta =\beta^{*}=\frac{\mu (\theta +\epsilon +\mu )(u_{1}+k_{1}+d_{1}+\mu )}{\delta (1-u_{2}\xi )(p\theta +q\theta +q\epsilon +q\mu )}. \end{array}$$

Linearization system of equation (E1) at DFE with $\beta =\beta^{*}$ as follows:

$$\begin{array}{r} A=\left[\begin{array}{ccccc} -\mu & 0 & -\frac{\left(1-u_{2}\xi \right)\left(\mu +d_{1}+k_{1}+u_{1}\right)\left(\theta +\epsilon +\mu \right)}{\left(\left(\theta +\epsilon +\mu \right)q+p\theta \right)\left(1-u_{2}\xi \right)} & 0 & 0\\ 0 & -(\theta +\epsilon +\mu ) & \frac{\left(1-u_{2}\xi \right)p\left(\mu +d_{1}+k_{1}+u_{1}\right)\left(\theta +\epsilon +\mu \right)}{\left(\left(\theta +\epsilon +\mu \right)q+p\theta \right)\left(1-u_{2}\xi \right)} & 0 & 0\\ 0 & \theta & \frac{\left(1-u_{2}\xi \right)q\left(\mu +d_{1}+k_{1}+u_{1}\right)\left(\theta +\epsilon +\mu \right)}{\left(\left(\theta +\epsilon +\mu \right)q+p\theta \right)\left(1-u_{2}\xi \right)} & 0 & 0\\ 0 & \epsilon & u_{1} & -(\mu +d_{2}+k_{2}) & 0\\ 0 & 0 & k_{1} & k_{2} & -\mu \end{array}\right]. \end{array}$$

From the Jacobian matrix, we get a simple zero eigenvalue (with all other eigenvalues having negative real parts). Hence, we can continue our step by computing the right eigenvector *w* of matrix A that correspondents to λ=0 , which satisfied Aw=λw. After that, we have:

$$\begin{array}{rl} w_{1} & =-\frac{\left(\mu +k_{2}+d_{2}\right)\left(\theta +\epsilon +\mu \right)\left(\mu +d_{1}+k_{1}+u_{1}\right)}{\left(\left(\left(p+q\right)u_{1}+p\left(\mu +d_{1}+k_{1}\right)\right)\epsilon +\left(p\theta +q\left(\mu +\theta \right)\right)u_{1}\right)\mu }<0, \end{array}$$

$$\begin{array}{rl} w_{2} & =\frac{p\left(\mu +k_{2}+d_{2}\right)\left(\mu +d_{1}+k_{1}+u_{1}\right)}{\left(\mu q+\left(\epsilon +\theta \right)\left(p+q\right)\right)u_{1}+p\epsilon \left(\mu +d_{1}+k_{1}\right)}>0, \end{array}$$

$$\begin{array}{rl} w_{3} & =\frac{\left(\mu +k_{2}+d_{2}\right)\left(\left(\theta +\epsilon +\mu \right)q+p\theta \right)}{u_{1}\left(\theta +\epsilon +\mu \right)q+\left(\left(\mu +d_{1}+k_{1}+u_{1}\right)\epsilon +\theta u_{1}\right)p}>0, \end{array}$$

$$\begin{array}{rl} w_{4} & =1>0, \end{array}$$

$$\begin{array}{rl} w_{5} & =\frac{\left(\left(\mu +k_{2}+d_{2}\right)k_{1}+k_{2}u_{1}\right)\left(\theta +\epsilon +\mu \right)q+\left(\left(\epsilon k_{2}+\theta \left(\mu +k_{2}+d_{2}\right)\right)k_{1}+k_{2}\left(\left(\mu +d_{1}+u_{1}\right)\epsilon +\theta u_{1}\right)\right)p}{\left(u_{1}\left(\theta +\epsilon +\mu \right)q+\left(\epsilon k_{1}+\left(\mu +d_{1}+u_{1}\right)\epsilon +\theta u_{1}\right)p\right)\mu }>0. \end{array}$$

Next, compute the left eigenvector *v* of matrix A that correspondents to λ=0 , which satisfied vA=vλ , then we have:

$$\begin{array}{r} v_{1}=0,\;v_{2}=1>0,\;v_{3}=\frac{\theta +\epsilon +\mu }{\theta }>0,\;v_{4}=0,\;v_{5}=0. \end{array}$$

The next step is calculating the value of a and b as follows:

– as we know that $v_{1}=v_{4}=v_{5}=0,$ then $g_{1},g_{4}$ and $g_{5}$ are not considered in calculation. Therefore, we just consider $g_{2}$ and $g_{3}$ in this calculation. Hence, we have the bifurcation coefficient a given by:

$$\begin{array}{l} a=\sum_{k,i,j=1}^{5}v_{k}w_{i}w_{j}\frac{\partial^{2}g_{k}}{\partial x_{i}\partial x_{j}}(E_{1},\beta^{*}),\\ \;\;=\sum_{i,j=1}^{5}v_{2}w_{i}w_{j}\frac{\partial^{2}g_{2}}{\partial x_{i}\partial x_{j}}(E_{1},\beta^{*})+\sum_{i,j=1}^{5}v_{k}w_{i}w_{j}\frac{\partial^{2}g_{3}}{\partial x_{i}\partial x_{j}}(E_{1},\beta^{*}),\\ \;\;=-\frac{2(p\theta +q\theta +q\epsilon +q\mu ){\left(\mu +d_{1}+k_{1}+u_{1}\right)}^{2}{\left(\theta +\epsilon +\mu \right)}^{2}{\left(\mu +k_{2}+d_{2}\right)}^{2}}{\delta {\left(\mu p\epsilon +\mu qu_{1}+p\theta u_{1}+p\epsilon d_{1}+p\epsilon k_{1}+p\epsilon u_{1}+q\theta u_{1}+q\epsilon u_{1}\right)}^{2}\theta }<0; \end{array}$$

– note that $v_{1}=v_{4}=v_{5}=0,$ then $g_{1},g_{4}$ and $g_{5}$ are not considered in this calculation. Hence, the bifurcation coefficient b is given by:

$$\begin{array}{l} b=\sum_{k,i=1}^{5}v_{k}w_{i}\frac{\partial^{2}g_{k}}{\partial x_{i}\partial \beta }(E_{1},\beta^{*}),\\ \;\;=\sum_{i=1}^{5}v_{2}w_{i}\frac{\partial^{2}g_{2}}{\partial x_{i}\partial \beta }(E_{1},\beta^{*})+\sum_{i=1}^{5}v_{3}w_{i}\frac{\partial^{2}g_{3}}{\partial x_{i}\partial \beta }(E_{1},\beta^{*}),\\ \;\;=\frac{\left(\theta +\epsilon +\mu \right)\left(\mu +k_{2}+d_{2}\right)(p\theta +q\theta +q\epsilon +q\mu )\left(1-u_{2}\xi \right)q\delta }{\theta \left(u_{1}\left(\theta +\epsilon +\mu \right)q+\left(\left(\mu +d_{1}+k_{1}+u_{1}\right)\epsilon +\theta u_{1}\right)p\right)\mu }\\ \;\;\;+\frac{\left(\mu +k_{2}+d_{2}\right)(p\theta +q\theta +q\epsilon +q\mu )\left(1-u_{2}\xi \right)p\delta }{\left(u_{1}\left(\theta +\epsilon +\mu \right)q+\left(\left(\mu +d_{1}+k_{1}+u_{1}\right)\epsilon +\theta u_{1}\right)p\right)\mu }>0. \end{array}$$

Because a is negative and b is positive, then by Catillo-Chavez & Song theorem equations (2.1)–(2.5) exhibits the phenomena of forward bifurcation at $R_{c}=1$ .

## Appendix F. Numerical experiments on bifurcation diagram and autonomous simulation

Here in this section, we performed the sensitivity analysis on the bifurcation diagram of equations (2.1)–(2.5) with respect to control parameters $u_{1}$ and $u_{2}$ and the infection rate β . The results are displayed in figures 10–12. The parameter values employed for numerical simulations in this section correspond to the optimized values for Indonesia, detailed in table 1, unless explicitly specified otherwise.

Figure 10*a* illustrates the monotonic decrease of the endemic equilibrium (red curve) and the control reproduction number (cyan curve) with increasing of $u_{1}$ . The endemic equilibrium persists until reaching the branching point (BP) at $R_{c}=1$ (i.e. $u_{1}=1.438$ ). Before the BP, the TB-free equilibrium is unstable (blue dotted line), while the TB-endemic equilibrium is stable (red solid curve). Once $u_{1}$ surpasses the BP, the endemic equilibrium ceases to exist, and the stability of the TB-free equilibrium shifts from unstable to stable. To illustrate the impact of $u_{1}$ on the dynamics of $I_{1},I_{2}$ and S , we specifically selected two sample points, namely $P_{1}$ ( $u_{1}=0.5$ , $R_{c}=1.916$ ) and $P_{2}$ ( $u_{1}=1.8$ , $R_{c}=0.844$ ). Figure 10*b* depicts the solution of equations (2.1)–(2.5) on the $S-I_{1}-I_{2}$ plane for various initial conditions at sample point $P_{1}$ , while figure 10*c* dprovides the corresponding illustration for sample point $P_{2}$ .

The sensitivity of $R_{c}$ and equilibrium point size of $I_{1}$ with respect to $u_{2}$ is plotted in figure 11. We employed identical parameter values as those used for figure 10 , with the exception of setting $u_{1}=1$ , while allowing $u_{2}$ to remain a free parameter. In figure 11*a*, we can see that the intervention of $u_{2}$ can significantly affect the $R_{c}$ . Hence, a stable branch of TB-endemic equilibrium existed for $u_{2}<0.787$ , while an unstable branch was observed for $u_{2}>0.787$ . We selected two sample cases at points $P_{1}$ and $P_{2}$ with corresponding $u_{2}$ values 0.2 and 0.9, respectively (seefigure 11*a*), to illustrate the impacts of $u_{2}$ on the dynamics of $I_{1},I_{2}$ and S . At the sample point $P_{1}$ , the solution converges to the endemic equilibrium point, while at $P_{2}$ , the solution converges towards the TB-free equilibrium point. Trajectories for both cases are displayed infigure 11*b,c*, for various initial conditions.

For the last one-parameter sensitivity analysis, we show how $R_{c}$ and endemic size of $I_{1}$ behave as β changes. It can be seen that larger β will increase $R_{c}$ and the endemic size of $I_{1}$ . The bifurcation diagram is shown in figure 12*a*. From the figure, we can see that the TB-free equilibrium is locally stable for β<0.000011 . As β>0.000011 , the stable branch of TB-free equilibrium point is bifurcated into an unstable TB-free equilibrium and a stable endemic equilibrium. The dynamics of $I_{1}$ corresponding to sample points $P_{1}$ and $P_{2}$ are plotted in figure 12*b,c*, respectively. It is important to mention that the solutions tended to move towards the endemic equilibrium when β was larger than the BP, and the solutions converged towards the TB-free equilibrium when β was smaller than the BP.

## Appendix G. Proof of Theorem 6: characterization of the optimal control problem

Let

$$\begin{array}{l} H=\omega_{1}I_{1}+\omega_{2}I_{2}+\varphi_{1}u_{1}^{2}+\varphi_{2}u_{2}^{2}+\cdots \\ \;\;\;\;+\lambda_{1}\left(\delta -(1-u_{2}(t)\xi )\beta SI_{1}-\mu S\right)\ldots \\ \;\;\;\;+\lambda_{2}\left(p(1-u_{2}(t)\xi )\beta SI_{1}-(\theta +\epsilon +\mu )E\right)\ldots \\ \;\;\;\;+\lambda_{3}\left(q(1-u_{2}(t)\xi )\beta SI_{1}+\theta E-(u_{1}(t)+k_{1}+d_{1}+\mu )I_{1}\right)\ldots \\ \;\;\;\;+\lambda_{4}\left(\epsilon E+u_{1}(t)I_{1}-(\mu +d_{2}+k_{2})I_{2}\right)\ldots \\ \;\;\;\;+\lambda_{5}\left(k_{1}I_{1}+k_{2}I_{2}-\mu R\right). \end{array}$$

Differentiate Hamiltonian H with respect to each control variables yields

$$\frac{d\lambda_{1}}{dt}=-\frac{\partial H}{\partial S},\;\;\;\frac{d\lambda_{2}}{dt}=-\frac{\partial H}{\partial E},\;\;\;\frac{d\lambda_{3}}{dt}=-\frac{\partial H}{\partial I_{1}},\;\;\;\frac{d\lambda_{4}}{dt}=-\frac{\partial H}{\partial I_{2}},\;\;\;\frac{d\lambda_{5}}{dt}=-\frac{\partial H}{\partial R},$$

with its transversality condition $\lambda_{i}(t_{f})=0$ for i=1,2,3,4,5 .

The optimal condition of $u_{1}$ taken by solving $\frac{\partial H}{\partial u_{1}}=0$ with respect to $u_{1}$ . Combining it with the lower and upper bound gives us:

$$u_{1}^{*}=\left\{\min\left\{\max\left\{u_{1}^{\min},\frac{I_{1}(\lambda_{3}-\lambda_{4})}{2\varphi_{1}}\right\}\right\},u_{1}^{\max}\right\}.$$

Similarly, we have

$$u_{2}^{*}=\left\{\min\left\{\max\left\{u_{2}^{\min},\frac{\xi \beta SI_{1}(p\lambda_{2}+q\lambda_{3}-\lambda_{1})}{2\varphi_{2}}\right\}\right\},u_{2}^{\max}\right\}.$$

Hence the proof is completed.

## Appendix H. Forecasting of India, Lesotho and Angola tuberculosis cases

See: figures 13–15.

## Appendix I. Numerical results on optimal control simulation for different strategy

See: figures 7–9.
